# Molecular Insights and Emerging Strategies for Treatment of Metastatic Uveal Melanoma

**DOI:** 10.3390/cancers12102761

**Published:** 2020-09-25

**Authors:** Fabiana Mallone, Marta Sacchetti, Alessandro Lambiase, Antonietta Moramarco

**Affiliations:** Department of Sense Organs, Sapienza University of Rome, 00161 Rome, Italy; fabiana.mallone@uniroma1.it (F.M.); marta.sacchetti@uniroma1.it (M.S.); antonietta.moramarco@uniroma1.it (A.M.)

**Keywords:** uveal melanoma (UM), metastatic uveal melanoma (mUM), prognostication, adjuvant therapy, metastatic therapy, metastatic dormancy, liver-directed-therapies, immunotherapy, targeted-therapy, combined therapy

## Abstract

**Simple Summary:**

Around 50% of patients with uveal melanoma (UM) still develop metastatic disease. Despite recent advances in the diagnosis and prognosis of UM, improvements in overall survival have not been achieved. At present, there is no available standard of care for adjuvant and metastatic settings. The aim of our review article was to discuss the latest advances in understanding the molecular mechanisms underlying uveal melanoma and novel treatment options for metastatic disease. We provided a detailed analysis of the most recently published works in the Literature along with a number of ongoing clinical trials for adjuvant and metastatic treatment of uveal melanoma. New insights into the pathogenesis of UM and promising results from the study of innovative tailored therapies could offer viable opportunities for translating in clinical practice.

**Abstract:**

Uveal melanoma (UM) is the most common intraocular cancer. In recent decades, major advances have been achieved in the diagnosis and prognosis of UM allowing for tailored treatments. However, nearly 50% of patients still develop metastatic disease with survival rates of less than 1 year. There is currently no standard of adjuvant and metastatic treatment in UM, and available therapies are ineffective resulting from cutaneous melanoma protocols. Advances and novel treatment options including liver-directed therapies, immunotherapy, and targeted-therapy have been investigated in UM-dedicated clinical trials on single compounds or combinational therapies, with promising results. Therapies aimed at prolonging or targeting metastatic tumor dormancy provided encouraging results in other cancers, and need to be explored in UM. In this review, the latest progress in the diagnosis, prognosis, and treatment of UM in adjuvant and metastatic settings are discussed. In addition, novel insights into tumor genetics, biology and immunology, and the mechanisms underlying metastatic dormancy are discussed. As evident from the numerous studies discussed in this review, the increasing knowledge of this disease and the promising results from testing of novel individualized therapies could offer future perspectives for translating in clinical use.

## 1. Introduction 

Uveal melanoma (UM) is the most common intraocular malignancy. UM originates from melanocytes of the uveal tract of the eye, including the iris, ciliary body, and retinal choroid. Despite successful control of the primary tumor and the significant improvements in early identification of patients at risk of metastatic progression, metastatic disease still occurs frequently and is invariably lethal. In addition, to date, there is no available standard of care for the treatment of metastatic UM (mUM). Current treatment protocols are mainly adapted from cutaneous melanoma, although they differ in terms of clinical and genetic profile, and ocular melanoma is often excluded from most clinical trials. 

In recent decades, the increasing understanding of tumor genetics, biology, and immunology has allowed for a better insight into the pathogenesis of UM; as a consequence, several clinical trials have been performed to investigate novel therapeutic targets for the treatment of UM in an attempt to change the disease course. 

In this paper, we reviewed recent advances in diagnosis, prognosis, and innovative treatment options for UM in adjuvant and metastatic settings, and perspectives for translating in clinical practice. Special emphasis was directed towards the mechanisms underlying metastatic dormancy and related therapeutic applications, as promising preventative strategies for metastatic growth.

## 2. Epidemiology and Uveal Melanoma Characteristics

UM is a relatively rare malignancy accounting for 5.3% of all melanoma cases recorded in the USA [1]; however, it represents the most common primary intraocular cancer in adults. Among ocular melanomas, 85% originates from the uvea, 4.8% from the conjunctiva, and the remaining 10.2% from other sites [1]. UM mostly arises from melanocytes located in the choroid (90%) and to a lesser extent in the iris (4%) and ciliary body (6%) [2]. The annual overall incidence of UM remained stable in recent decades at approximately 5.1 cases per million individuals in the USA and between 1.3 and 8.6 cases per million in Europe [3,4,5,6,7,8,9,10]. The disease is more frequent in Caucasian ethnicity, with a median age of presentation of approximately 60 years and 30% greater incidence in males [3,4,5,7,11,12,13]. Most relevant predisposing factors for the development of UM are the presence of dysplastic nevus syndrome, choroidal nevi, ocular or oculodermal melanocytosis, familial syndromes including germline BAP1 (BRCA1-associated protein 1) mutations, and neurofibromatosis [14,15,16,17]. Of note, cutaneous melanoma is not a risk factor for UM [18,19]. One in 8000 choroidal nevi transform into melanoma. The elements suggestive of a malign lesion are listed in Table 1 [20,21,22]. The chance of transformation is 4% if any characteristic is present, and it is more than 50% if three or more features are combined [20,21]. Choroidal nevi that do not exhibit any malignant feature require initial monitoring twice a year and then once a year if stability persists. Those showing 1 or 2 features need strict monitoring at least every 4–6 months, while those with 3 or more features should be referred to a specialized center for possible primary treatment and prognostic stratification [20,21]. According to the Collaborative Ocular Melanoma Study (COMS), the diagnosis of UM can be exclusively clinical, with a clinical misdiagnosis rate of only 0.48% [23]. However, other studies highlight the importance of fine-needle aspiration biopsy (FNAB) for diagnostic accuracy in selected cases [24]. In recent decades, treatment of primary UM has been evolving from enucleation towards effective eye-conserving modalities inclusive of radiation, surgical, and laser therapy [25]. However, the five-year survival rate has not registered substantial improvements during the past four decades, and it is still estimated at 70–80% irrespective of the type of treatment [4,9,26,27,28,29,30]. Radiotherapy and surgery achieve local disease control exceeding 90%, but approximately 50% of patients ultimately develop metastases, with UM showing a considerably worse prognosis than its cutaneous counterpart [4,26,31,32,33]. The estimates of metastatic progression are reported from 32% at 5 years, to 50% at 15 years, and 62% at 35 years [26]. The uveal tract is rich in vascular structure, and UM is peculiar for its almost exclusive dissemination via the hematogenous route with a propensity for the liver as the first site of metastasis in over 90% of cases. However, conjunctival lymphatic infiltration following direct invasion of the sclera has also been described [34]. The main predictors of metastatic progression of UM include clinical (tumor thickness and basal diameter, ciliary body involvement, degree of extraocular extension), histopathologic (epithelioid cytomorphology, infiltrating lymphocytes and macrophages, fibrovascular loops and networks, high mitotic activity), and genetic factors [35,36,37,38,39,40].

## 3. Prognosis of UM

Recent studies showed that UM characterization based on cytogenetics and gene expression profiling (GEP) significantly improved the prognosis in UM [38,41,42,43,44,45]. As consequence, great efforts have been made to identify karyotype or gene alterations which are associated with higher tendency to metastatic spread. 

Based on the increased understanding of UM genetics, some authors proposed integrating the American Joint Committee on Cancer (AJCC) Tumor-Node-Metastasis (TNM) clinical staging system with genetic parameters to improve the prediction of metastatic progression [39,46,47,48,49,50]. These studies showed that the prognostic significance of tumor basal diameter was considerably enhanced when considered together with chromosome mutational profile and histological grade, leading to UM clustering into low/high-risk models from large cohort studies [39,46,47,50]. Likewise, the group of Vaquero-Garcia developed a model for personalized Prediction of Risk of Metastasis in UM (PRiMeUM) based on clinical and chromosomal information, showing 85% prediction accuracy [48]. Concomitantly, efforts were dedicated to the study of classifications solely based on genetic data [43,44,45,51]. Recently, The Cancer Genome Atlas (TCGA) classification has been proposed to improve the identification of high-risk patients for metastatic disease with respect to an AJCC-TNM clinical staging system [52]. Specifically, the TCGA project, starting in 2005, was designed to conduct an expression analysis of mRNA, micro RNA, and long noncoding RNA and catalog genetic mutations of 33 different cancer types, including UM [53]. Based on TCGA results of chromosome 3 disomy or monosomy and degree of chromosome 8q gain, UM was categorized into 4 classes (A, B, C, and D) of progressive worsening prognosis [51]. Tumor clinical features and outcomes of metastatic risk and death were evaluated in a large sample of UM categorized on the basis of TCGA system, and the more advanced classes corresponded to older age, greater tumor size, and worst prognosis [54]. 

### Cytogenetic Alterations in UM

The most studied karyotype alterations in UM include chromosome 3 and 8. Chromosome 3 complete monosomy is the most frequent karyotype abnormality observed in almost half of all UM [38,55,56,57]. The presence of this monosomy is associated with a five-year survival rate of 39%, whereas a 90% five-year survival rate is observed for UM without monosomy 3 [58]. Interestingly, BRCA1-associated protein-1 (BAP1) is a tumor-suppressor gene placed on chromosome 3, and it is mutated in 47% of primary UM and up to 91% of metastatic UM [59,60,61,62,63,64]. The splicing factor 3B subunit 1 (SF3B1) is consistent with disomy 3 and shows more favorable prognosis, although an association with delayed metastases has been reported [65,66,67]. Eukaryotic translation initiation factor 1A, X-linked (EIF1AX) gene mutations are also described along with SF3B1 in UM with disomy 3, but metastatic tendency is less frequent [68]. Among other chromosome alterations, rearrangements in chromosome 8q have been described in approximately 40% of UM. Specifically, UM with normal 8q profile have 93% five-year survival, while those with one additional copy have 67% and those with 8q amplification have 29% [58,69]. A recent study on 1059 UM patients over 8 years follow-up, showed that the concurrent presence of 3 complete monosomy and 8q gain resulted in an increased risk of metastasis and death [43]. Interestingly, the most severe mutational events consisting of chromosome 3 loss and chromosome 8q gain correlated positively with ciliary body involvement, tumor thickness and basal diameter, proximity to the optic disc, extraocular spread, epithelioid cells, and age [70]. This evidence suggests that early intervention, when tumor growth is limited and the genetic profile more favorable, could prevent tumor dedifferentiation into a more aggressive type [54,70]. Further cytogenetic aberrations associated with an increased risk of distant recurrences include 1p loss, 6q loss, and 8p loss [71,72]. Due to advances in knowledge on cancer biology, over the last two decades, genetic tests are routinely performed in clinical practice, ranging from single chromosome 3 evaluation to arrays of analyses for chromosomes 1, 3, 6, and 8 and GEP. However, both cytogenetic testing and GEP for prognosis of UM require invasive procedures to harvest the specimens from either enucleation or intraoperative FNAB [44,73,74,75,76]. Specifically, among cytogenetic tests, analysis of a karyotype using fluorescence in situ hybridization (FISH) and the array comparative genomic hybridization (aCGH) allows for the detection of translocations and partial deletions of the chromosome 3 and requires a large amount of tissue with a technical failure rate with FNAB of approximately 50% [77,78]. Among other genetic analyses, multiplex ligand-dependent probe amplification (MLPA) consists of 31 probes to analyze loci on chromosomes 1p, 3, 6, and 8 and, thus, to identify high- and low-risk patients. Similar to FISH and aCGH, MLPA requires large tissue samples with increased risk of biopsy complications [47]. An alternative cytogenetic method requiring a lower number of samples is represented by microsatellite analysis (MSA). This technique combines fluorescent probes with PCR, and has proven accurate for the identification of aberrations on chromosome 3, but not for those on chromosomes 8 and 6 [57]. Besides these limitations, cytogenetic tests are prone to sampling errors in UM due to its dense cellularity and elongated nuclei that weave in and out of the plane of section, and to significant intratumoral heterogeneity [47,79,80]. GEP, using an RNA-based assay, currently represents the gold standard in molecular prognosis. This test has a technical failure rate of only 3%, and can be performed on fine-needle biopsies even when the quantity of RNA is below detectable limits. It allows for the categorization of UMs in Class 1 and Class 2 based on low and high risk of metastatic potential, respectively [44,45]. The prognostic accuracy of GEP classification has proven superior over cytogenetic methods, and this would be related to the heterogeneous distribution of chromosomal markers throughout the tumor. GEP analysis is indeed very sensitive for detecting the proper class signature in heterogeneous tumors as it performs simultaneous evaluation of several genes representative of the tumor microenvironment [45]. There is evidence reporting that GEP is more capable of capturing the overall tumor functional complexity than a chromosomal marker [47,80]. The group of Onken et al. developed a clinically feasible platform for analyzing GEP by a 15-gene PCR-based assay [81]. This assay is now commercially available in the United States as DecisionDx-UM^®^ (Castle Biosciences, Friendswood, TX, USA), and it is highly accurate, easy to interpret, and independent from additional analyses. In detail, GEP Class 1 is subdivided into class 1A and class 1B, with 2 and 21% five-year metastatic risk, respectively, whereas GEP Class 2 is associated with a five-year metastatic risk of 72% [66,82]. Recently, GEP classification has been revised based on PRAME (preferentially expressed antigen in melanoma) status. PRAME has been reported as an independent prognostic biomarker for UM that identifies increased metastatic risk in patients with Class 1 or disomy 3 tumors. When combined with a 12-gene expression panel, PRAME messenger-RNA expression predicted a five-year metastatic rate of 0 in class 1/PRAME−, 38% in class 1/PRAME+, and 71% in class 2 tumors. Interestingly, PRAME+ status was positively correlated with larger tumor diameter after analysis of the TCGA Research Network dataset. PRAME expression positively correlated with larger tumor diameter and SF3B1 mutations as well as gain of 1q, 6p, 8q, and 9q and loss of 6q and 11q [83].

## 4. Adjuvant Therapies and Surveillance of UM

Despite significant advances in prognosis and identification of high-risk patients, adjuvant systemic treatments effective in preventing metastases or improving outcomes in UM are not yet available in clinical practice [84,85]. Therefore, intensified surveillance appears crucial for the early detection of oligometastatic disease manageable with liver-directed therapies, as well as to enroll patients eligible for clinical trials [86]. Specifically, patients identified at a high-risk of metastatic progression based on cytogenetics or GEP should have six-monthly life-long surveillance including a clinical review, nurse specialist support, and liver-specific imaging by a nonionizing modality [87]. It is reported that magnetic resonance imaging (MRI) obtained every six months is capable of detecting the metastases before the onset of symptoms in 92% of cases [86]. A few adjuvant therapy trials have been tested in UM based on favorable results in cutaneous melanoma. A randomized controlled clinical trial investigated the effects of dacarbazine (DTIC)—an intravenous alkylating agent—demonstrating no survival advantage over observation in UM [88]. Likewise, a randomized study on methanol-extraction residue of bacille Calmette-Guérin (BCG) reported no benefit in improving the survival rate [89]. Two nonrandomized studies failed to demonstrate beneficial effects on survival rate with systemic adjuvant low-dose interferon-alpha (IFN-α) compared with matched historical controls [90,91]. Fotemustine, an alkylating agent with high hepatic uptake, has been studied as an adjuvant therapy for UM by intra-arterial hepatic delivery, with a trend towards improved survival but not statistical significance compared with matched historical controls [92]. However, these adjuvant studies were conducted before the introduction of molecular methods of prognosis. In this respect, a multicenter randomized phase III clinical trial (FOTEADJ) based on genomic analysis in high risk UM patients treated with adjuvant fotemustine versus observation was performed, but it was stopped earlier for futility [93]. Similarly, a nonrandomized prospective phase II clinical trial designed to evaluate sequential low-dose DTIC and IFN-α-2b in cytogenetic high-risk patients was completed, but it failed to reach the primary outcome of progression free survival (PFS) or overall survival (OS) increase at five-year follow-up [94]. Novel classes of molecules have been investigated in the adjuvant setting for UM with promising results. The tyrosine-kinase receptors c-Met and c-Kit are highly expressed in UM and activate the Ras/Erk, and PI3-kinase pathways following binding to the hepatocyte growth factor (HGF) and stem cell factor (SCF), respectively. These pathways have been definitely demonstrated to be involved in cancer occurrence and progression [95]. Crizotinib—a tyrosine-kinase inhibitor that inhibits the phosphorylation of c-Met—was shown to significantly reduce the development of distant metastases in a murine model of metastatic UM when compared with an untreated control group [96]. Of note, crizotinib is currently under evaluation in patients with UM [97]. A retrospective cohort study based on adjuvant sunitinib—a tyrosine-kinase inhibitor that inhibits c-Kit—and conducted in high-risk patients stratified according to cytogenetics and GEP, resulted in three- and five-year improvement of OS estimates [98]. To confirm such results, a phase II pilot clinical trial evaluating sunitinib, tamoxifen, and cisplatin in patients with high-risk ocular melanoma is ongoing [99]. Similarly, a randomized, noncomparative phase II clinical trial investigating sunitinib and the histone deacetylase (HDAC) inhibitor, valproic acid, for high-risk tumors in an adjuvant setting is currently ongoing [100]. The assumption for using HDAC inhibitors in the adjuvant setting for UM is based on their ability to reverse the phenotypic effects of BAP1 loss in cultured UM cells [101,102]. Another interesting approach for the treatment of UM is the use of adjuvant dendritic cell (DC) vaccination. An open-label phase II clinical trial was performed to investigate immunologic responses after adjuvant DC vaccination in patients defined at high-risk based on cytogenetics. This study showed an increase in OS in patients with a detectable tumor antigen-specific immune response [103]. In addition, a multicenter, randomized, two-armed, open-label phase III study to evaluate the adjuvant vaccination with tumor RNA-loaded autologous DCs in patients with resected monosomy 3 UM is currently ongoing [104]. A similar phase I/II study on mRNA transfected autologous DCs in high-risk uveal melanoma patients has recently been closed due to slow accrual [105]. Immunotherapy with monoclonal antibodies, such as antiprogrammed cell death protein 1 (PD-1) nivolumab, anticytotoxic T-lymphocyte-associated Protein 4 (CTLA-4) ipilimumab, and anti lymphocyte activation gene 3 (LAG-3) relatlimab, modulated the immune responses in the tumor microenvironment and interfered with tumor growth and spread [106,107]. A small sample size phase I/II pilot trial of adjuvant ipilimumab in high-risk primary uveal melanoma demonstrated that 80% of patients were disease-free at 36 months [108]. A randomized phase II trial, evaluating nivolumab with or without ipilimumab or relatlimab in neoadjuvant and adjuvant settings, is currently ongoing in patients with AJCC stage IIIB–IV posterior UM [109]. Similarly, a phase II single-arm multicenter study to evaluate the effects of adjuvant ipilimumab treatment in combination with nivolumab in subjects with high-risk UM is currently recruiting [110]. An innovative approach is represented by prophylactic radiation therapy to the liver; however, a phase II trial on external-beam hepatic irradiation in high-risk patients has recently been closed for lack of accrual [111]. Moreover, there is recent evidence of potent antitumor effects in UM cells following nonselective beta-blocker administration, and concurrent expression of β1 and β2 adrenoceptors in UM specimens. These findings suggest that further investigation is needed in the context of clinical trials for adjuvant scope [112]. Current clinical trials in the adjuvant setting are summarized in Table 2.

## 5. Metastatic Dormancy and Therapeutic Opportunities

A proportion of only 1–2% of patients with UM presents metastatic disease at the time of diagnosis [113,114]. However, mathematical models of cell doubling times and direct histopathologic evaluation suggest that UM hepatic micrometastases would be present since the time of initial diagnosis [115,116,117,118]. The identification of circulating tumor cells in the bloodstream of patients clinically free of metastasis supports this consideration [117,119]. It is worth noting that early detection and primary treatment showed to have an impact on disease-related morbidity but not on patients’ OS [7,118,120,121]. Moreover, there is evidence that UM liver metastases may remain stable for years until an exponential proliferation occurs [122]. Based on these observations, a growing interest has been focused in the study of the mechanisms underlying UM cell dormancy in the liver and potential ways to prolong or specifically target them [115,116,117,118]. The identification of the key factors involved in the transition to rapidly growing tumors is crucial for the development of novel therapeutic strategies in order to prolong metastatic dormancy or eliminate dormant cancer cells in a controlled fashion. In fact, the state of dormancy confers refractoriness to conventional therapies aimed at targeting rapidly proliferating cells. Several studies have been performed to identify novel therapeutic targets able to control or eliminate metastatic dormant cells in different cancers; however, metastatic dormancy has not yielded appropriate clinical investigation in melanoma, and specifically in UM. Dormant tumor cells are supposed to be in a quiescent state, prevented from proliferating exponentially due to blockage of the cell cycle. The dormancy of disseminated tumor cells is supposed to be the result of a balance between anti- and protumorigenic immune and inflammatory responses, failure in activating the angiogenic switch, genetic modulation by metastasis suppressor genes (MSGs), and associated signaling pathways [123,124,125]. The arrest of circulating cancer cells adhering to the sinusoidal endothelium of the hepatic lobules leads to avascular micrometastasis development in periportal areas [126]. Simultaneously, tumor lytic M1 phenotype macrophages are recruited along with activated hepatic stellate cells (HSCs) secreting extracellular matrix and proinflammatory mediators [126,127,128]. The paracrine signaling on UM cells would then lead to a reduction in D-type cyclin and a deregulation in the interaction of the cyclin-dependent kinases (CDKs) with the CDK inhibitors (CKIs), with consequent arrest of UM cells proliferation [129,130]. The potential to evade the immune system in cancer is associated with increasing tumor genetic instability and related reduction in immunogenicity following progression [119,122]. In accordance, CD4+ tumor infiltrating lymphocytes (TILs) and CD8+ TILs have been described in advanced metastatic disease with perivascular and peritumoral distribution, respectively. Thus, suggesting their inability to infiltrate the tumor mass as disease progresses [127,131,132]. Furthermore, a clear prevalence of proangiogenic and protumorigenic M2 phenotype macrophages has been demonstrated within hepatic mUM in late stages of the disease [127,131]. Interestingly, BAP1 loss has recently been correlated with upregulation of several genes associated with suppressive immune responses and, at the protein level, with entrapment of infiltrating immune cells within peritumoural fibrotic areas surrounding mUM [62]. There is evidence suggesting that dormant cells express mutant neoantigens, which can occur naturally or rather derive from treatment [133,134]. The low frequency of naturally occurring neoantigen-specific T cells clones, anyway, has favored the advent of specific adoptive T-cells transfer therapies [135,136]. Adoptive T-cells transfer therapies may be of interest for UM since the primary tumor originates in the immune-privileged environment of the eye, and it may present tumor neoantigens for which the host’s immune system is not prepared. T-cells targeting glycoprotein gp100 were tested for this scope in vitro and in vivo in human UM, demonstrating homing to the eye and effective tumoricidal function [137]. Recently, cell-based vaccines modified to express MHC II alleles syngeneic to the recipient and the costimulatory molecule CD80 have been studied in UM. These vaccines were capable of activating CD4+ T cells specific to uveal melanoma neoantigens, that in turn reacted with primary UM cells and cross-reacted with mUM cells. Moreover, CD4+ T cells activated CD8+ T cell-mediated immunity against primary and mUM cells [138]. In addition, IFN-γ production by CD4+ T cells activated by UM vaccines, promoted an antitumor response by inhibiting neovascularization and tumor cell proliferation, as well as upregulating tumor-expressed MHC molecules [138]. In detail, IFN-γ showed the ability to mediate long-term cell growth arrest in vitro and in vivo nude mice models via STAT1 and p27-dependent mechanisms, and it also raised the hypothesis of inducing specific T-cells capable of IFN-γ production upon recognition of tumor cells [139,140,141]. For this purpose, a phase I/II clinical trial to evaluate the efficacy of immunotherapy with TILs in combination with intratumoral injections of IFN-γ-adenovirus in cutaneous metastatic melanoma is ongoing [142]. Moreover, a study investigating tumor microenvironment, demonstrated that the use of low doses of the anti-VEGF receptor 2 (VEGFR2) antibody was able to polarize the immune inhibitory M2-like phenotype towards the immune stimulatory M1-like phenotype and to recruit CD4+ and CD8+ T-cells. These mechanisms suggested that low-dose antiangiogenic treatment in adjunct to vaccine therapy could enhance anticancer efficacy [143]. Among other factors regulating the awakening of dormant cells, the angiogenetic sprout allowing the shift from prevascular to highly vascularized lesions is known to play a critical role [123]. The group of Grossniklaus et al. identified pseudo-sinusoidal spaces between the sinusoidal endothelium and hepatocytes (space of Disse), developing from stellate cells to nourish large infiltrative pattern metastases [144,145]. The hepatic fibrosis/stellate cell activation and the mTORC1/S6K signaling axis have been fully characterized while profiling secretome from high-risk metastatic UM compared to normal choroidal melanocytes [146]. In a study on GEP analysis in experimental animal models of different human types of cancer, a downregulation of angiogenesis inhibitor thrombospondin and decreased sensitivity to angiostatin in switched fast-growing versus dormant tumors were described [147]. Other genes associated with the angiogenic process were observed to contribute to tumor dormancy, including tropomyosin, transforming growth factor beta 2 (TGF-b2), Eph receptor A5 (EphA5), histone H2BK, proline 4-hydroxylase alpha polypeptide I, and insulin-like growth factor binding protein 5 (IGFBP-5) [123]. Several studies not including UM investigated the role of MSGs in preventing the formation of metastases and favoring dormancy [124,125,141,148,149,150,151,152,153,154,155,156,157,158,159]. Specifically, the MSGs KISS1, RhoG-DI2, and Nm23-H1 showed to be able to suppress the development of distant metastases without significantly affecting tumor growth at the primary site [124,125]. Interestingly, MSGs rarely mutate, and their downregulation in highly metastatic tumors would rather be associated with epigenetic modifications. In this regard, possible therapeutic targets could be represented by DNA methyl-transferases and histone deacetylases [102,160,161]. 

## 6. Treatment of Metastatic Disease

UM patients with metastatic disease are hardly candidates for curative treatments, with a reported 15% one-year survival and an average life expectancy varying in literature from 6 to 12 months [1,2,6,26,30]. There is currently no standard of care for the treatment of mUM, and available treatments are mostly adapted from cutaneous melanoma protocols in spite of their different clinical and genetic profiles [162]. Furthermore, patients presenting with ocular or mucosal melanoma are frequently excluded from clinical trials. Thus far, systemic chemotherapy has provided poor response rates (0–15%) in clinical trials for UM metastatic disease [163,164,165]. Liver-directed therapies have shown limited improvements in response rates, but no benefit in OS [166,167,168,169]. Unlike the positive results achieved in the treatment of metastatic cutaneous melanoma, immunotherapy and targeted therapies have failed to improve OS in metastatic UM. 

### 6.1. Chemotherapy 

Systemic chemotherapy, adopted from cutaneous melanoma, has been evaluated in the context of single-arm phase II studies in mUM as monotherapy, including dacarbazine, temozolomide, fotemustine, and docosahexaenoic acid (DHA)-paclitaxel, or combined treatments (BOLD regimen+interferon α-2b, dacarbazine and treosulfan or cisplatin, gemcitabine and treosulfan, cisplatin, dacarbazine and vinblastine). Similar response rates of less than 15%, PFS limited to 4 months, and OS of no more than 12 months were reported [163,164,165,170,171,172,173]. Moreover, significant hematological, pulmonary and neurological toxicities were observed. Therefore, research is rather addressed to the development and testing of targeted and immune therapies, as well as liver locoregional approaches.

### 6.2. Liver-Directed Therapies 

The liver is the first and the sole site of metastatic spread in more than 50% of mUM patients, and liver-directed therapies including surgical resection, regional perfusion, and embolization have been investigated in patients with mUM confined to the liver [26]. Available knowledge on the efficacy of liver-directed therapies is mostly based on down-sized, retrospective, single-institution studies. A recent meta-analysis determining benchmarks of PFS and OS for mUM suggested more favorable outcomes with liver-directed therapies compared with chemotherapy, immunotherapy, and targeted therapy, even after adjusting for prognostic factors [174]. 

#### 6.2.1. Surgery

It is reported from retrospective cohort studies that surgical resection of oligometastases is effective for curative intent in mUM. Curative (R0) resection is the most important positive prognostic factor following liver resection [87]. However, only 5–10% of patients are candidates for surgery based on liver metastase distribution and size [175,176,177]. Comparative studies in patients with mUM showed that median OS ranges between 10 and 35 months in patients treated with surgery (differing between R0 resection or debulk of metastases), between 9 and 15 months with any systemic treatment, and between 2 and 6 months with the best supportive care, from comparative studies on mUM [86,121,175,176,177,178,179,180,181,182,183]. In the largest series currently available, longer survival was associated with metastasis-free intervals longer than 24 months; R0 resection, number of liver metastases ≤ 4, and absence of miliary disease were associated with prolonged survival [175]. The group of Akyuz et al. demonstrated five-year survival exceeding 20% after complete tumor destruction under laparoscopic resection or laparoscopic radiofrequency ablation (RFA) including a nonsurgical comparator group [177]. Confirming rates were reported in several noncomparative studies [176,182,183,184,185,186]. Other studies evaluated RFA or hepatic intra-arterial chemotherapy (HIA) as an adjunct to liver surgery to increase the number of patients with bilobar metastases achieving R0 resection [181,187,188]. Importantly, RFA associated with liver surgery and liver surgery alone demonstrated similar survival outcomes [187]. In addition, a recent study evaluated combined surgery and RFA in a small group of patients relapsing after complete first liver resection, showing prolonged survival outcomes [189]. However, postresection local and distant recurrences are frequent exhibiting rates of 75%, and there are no data from randomized clinical trials which demonstrate survival benefit over systemic therapy [183,186]. 

#### 6.2.2. Regional Perfusion Therapies

Direct targeting of hepatic arterial circulation represents an attractive strategy for unresectable isolated liver disease. Metastases to the liver are indeed preferentially supplied by hepatic artery branches unlike normal hepatic circulation, receiving blood mainly from the portal vein. Regional approaches allow the direct delivery of high doses of chemotherapy with minimal systemic exposure and include hepatic intra-arterial chemotherapy (HIA), isolated hepatic perfusion (IHP), percutaneous hepatic perfusion (PHP), and hepatic transarterial chemoembolization (TACE). A phase III randomized clinical trial from the European Organization for the Research and Treatment of Cancer (EORTC) assigned 171 patients with UM and liver metastases to receive fotemustine via HIA or intravenously (IV). Significant improvements were registered in PFS (4.5 vs. 3.5 months) and response rate (10.5 vs. 2.4%) with HIA compared with IV administration, but no difference was demonstrated between the two arms in terms of OS (median 14.6 months for HIA vs. 13.8 months for IV fotemustine) [166]. IHP, as open or percutaneous procedure (PHP), is a form of intra-arterial chemotherapy requiring a temporary extracorporeal filtration system to surgically isolate the liver from systemic circulation. Results from a phase II clinical trial suggested a survival advantage of 14 months for patients treated with IHP using melphalan compared with the longest survival rate of patients with UM liver metastases not treated with IHP, associated with tolerable morbidity [190]. The SCANDIUM study—a randomized multicenter phase III clinical trial—is currently ongoing in patients with UM and isolated liver metastases to evaluate the efficacy of IHP melphalan compared with the best alternative care in OS [191]. Results from a randomized phase III trial including 93 patients with melanoma metastatic to the liver (88% ocular, 12% cutaneous) treated with either PHP with melphalan or best available care—showed that PHP was effective in significantly improving median PFS (245 days vs. 49 days, *P* < 0.001) and overall response rate (34.1 vs. 2% *P* < 0.001). This study failed to demonstrate survival overall benefit; however, the crossover design of the study may confound the survival data [167]. The phase III multicenter FOCUS clinical trial is currently ongoing in patients with metastatic disease and hepatic-dominant UM treated with either PHP with melphalan or distinct options under the best alternative care (transarterial chemoembolization, dacarbazine, ipilimumab, or pembrolizumab)—randomly assigned. However, due to accrual issues, this study was later modified to remove randomization [192]. Another strategy among liver-locoregional treatments for mUM is TACE with infusion of chemotherapeutics including cisplatin, carboplatine, mytomicin, fotoemustine, and 1,3-bis (2-cholorethyl)-1-nitrosourea (BCNU), followed by embolization agents such as iodized oil or polyvenylalcohol particles. In a large, retrospective cohort study, chemoembolization was effective when compared with systemic therapies in inducing 33% response rate versus 1%; however, no survival benefit was demonstrated [193]. Similar findings were reported in noncomparative studies, with overall response rates varying from 20.4% to 46% [193,194,195,196,197,198]. From a phase II study, improved response rates and survival were demonstrated in patients with less than 20% liver involvement, suggesting that small and well demarcated tumors receiving their supply solely from the hepatic artery represent the best targets for embolization [196]. In accordance, other studies reported that an extent of liver involvement > 50% predicted poor outcomes with arterial chemoembolization [197]. Importantly, two-thirds of patients with stabilization of hepatic metastases following TACE developed dissemination in extrahepatic sites within a short time, thus raising the issue of combining systemic immuno-chemotherapy with local treatments [196]. In a pilot clinical trial, platinum-based TACE with polyvenylalcohol (PVA)-particle embolization in combination with systemic immuno-chemotherapy achieved a 57% partial response rate and survival benefit [199]. A phase-I/II randomized trial evaluated HIA with cisplatin and TACE with cisplatin and polyvinyl sponge (PVS) in 19 patients with UM metastatic to the liver, reporting a modest overall response rate (16%) and dose-limiting toxicities [200].

#### 6.2.3. Radioembolization 

Among other techniques, radioembolization (RE) using yttrium-90 (^90^Y)-labeled microspheres was evaluated as salvage therapy in the context of small, retrospective cohorts, reporting median OS rates ranging from 9 to 24 months. Partial response or a stabilization of the disease was reported for 57 and 77% of patients, respectively [168,169]. Recently, a prospective phase II clinical trial evaluated the efficacy of RE in treatment-naïve patients with mUM (group A) and in participants who progressed after immunoembolization (IE) (group B). This study demonstrated similar median OS (18.5 months and 19.2 months) and 1-year survival rate (60.9 and 69.6%) between the two groups, respectively. Interestingly, the stabilization of hepatic disease was achieved in 87.0% of participants in groups A versus 58.3% in group B [201]. In a recent single arm, open labeled, nonrandomized study, the combination of yttrium-90 microspheres and intravenous cisplatin was well tolerated in mUM, but it failed at demonstrating sustained disease control with a median PFS of 3 months and median OS of 10 months [202]. A nonrandomized phase I clinical trial investigating ^90^Y -labelled microspheres in combination with sorafenib—a multikinase inhibitor of cell proliferation and angiogenesis—was concluded, but the results have not yet been published [203].

#### 6.2.4. Immunoembolization (IE)

The increased release of tumor antigens after tumor destruction via embolization leads to the development of immunoembolization (IE) using granulocyte-macrophage colony-stimulating factor (GM-CSF). A randomized phase II study, investigating IE versus bland embolization (BE) in patients with mUM, demonstrated similar OS rates (21.5 months in IE group versus 17.2 months in BE group), with a significant survival advantage in patients with at least 20% of liver involvement within the IE cohort. Moreover, the intense inflammatory reaction in response correlated positively with delayed progression of extrahepatic metastases [204].

### 6.3. Immunotherapy 

Immunological checkpoint inhibitors targeting the cytotoxic T-lymphocyte associated antigen (CTLA)-4 (ipilimumab), the programmed cell death 1 (PD)-1 protein (pembrolizumab, nivolumab), or the programmed cell death 1 ligand (PDL)-1 (durvalumab, atezolizumab) aim at stimulating endogenous antitumor cytotoxic T cell response. The efficacy achieved in the management of metastatic cutaneous melanoma and other cancers with reported durable response rates ranging from 20 to over 60%, has not been observed in mUM [205,206,207]. Studies reported a response rate below 10%, and a median survival less of than 1 year with a single-agent checkpoint block have been widely described [208,209,210,211,212,213]. This is likely related to the immune privilege of the eye, which establishes mechanisms to evade the immune system, and with the low mutational load with limited potential neoepitopes of UM if compared with cutaneous melanoma. However, clinical benefit from PD-1 block has been reported in selected UM patients with biallelic MBD4 loss showing a high mutational burden [214]. Two phase II clinical trials are investigating combinatorial checkpoint blockade with nivolumab and ipilimumab in treatment-naïve or pretreated patients with mUM [215,216]. Specifically, preliminary results from the clinical trial NCT01585194 showed a partial remission (PR) in 17% of patients, and a stable disease in 53% of patients. The median OS was estimated at 1.6 years, and the 1-year OS was 62%. However, 40% of patients experienced treatment-related adverse events (TRAEs), with 29% of treatment discontinuation [217]. A multicenter phase II open label study evaluating the concomitant use of pembrolizumab and entinostat (HDAC inhibitor) in adult patients with metastatic mUM (PEMDAC study) is currently ongoing [218,219]. A phase Ib/II clinical trial demonstrated that combination treatment with RFA and ipilimumab in uveal melanoma (SECIRA-UM) was well tolerated, but with very limited clinical activity [220]. Importantly, a randomized phase I/II study is currently ongoing in mUM patients to evaluate the safety and efficacy of combining melphalan PHP with ipilimumab and nivolumab [221]. In addition, the efficacy of the combination of ipilimumab and nivolumab has been investigated in association with IE, and following ^90^Y radioembolization, respectively, in two ongoing phase II trials [222,223]. Novel immune-based therapies include different modalities of adoptive T cell therapy such as TILs, engineered T cell receptors (TCRs) and chimeric antigen receptors (CAR) on T cells, and T cell redirection [224,225,226,227]. Adoptive transfer of autologous TILs has shown promise in mediating tumor regression in a single-center phase II study on refractory mUM patients who showed progression after both anti-CTLA-4 and anti-PD-1 checkpoint blockade [224]. A phase II study on immunotherapy using autologous TILs in mUM is currently ongoing [228]. In this direction, a phase Ib study combining adoptive T cell therapy using autologous CD8+ antigen-specific T cells and anti-CTLA-4 for patients with mUM is ongoing [229]. IMCgp100 is a bispecific ImmTAC (Immune-mobilizing monoclonal TCRs against cancer) molecule, targeting gp100 peptide on UM cells and the CD3 protein complex on the surface of T cells. It redirects the recruitment of CD8+ cytotoxic T lymphocytes against melanoma cells. This molecule has shown a favorable safety profile and durable responses in mUM, and it is currently under investigation in advanced UM in a single-arm, phase I/II dose-escalating clinical trial and in a randomized, controlled phase II trial versus the investigator’s choice of therapy [230,231,232,233]. As an additional promising strategy, CAR-T cells directed against human epidermal growth factor receptor 2 (HER2) were demonstrated to be able to kill uveal and cutaneous melanoma cells in vitro and in vivo settings [226]. A phase I clinical trial is evaluating the effect of autologous T-lymphocytes expressing GD2-specific chimeric antigen on different GD2-expressing cancers including UM [234]. Among immunotherapeutic options for mUM, cell-based and peptide vaccines are currently being investigated in several ongoing clinical trials as single therapy or in combination with immunomodulatory agents, based on favorable preclinical and clinical studies [138,235,236,237,238]. As innovative approach, liver intralesional PV-10 chemoablation (10% rose bengal disodium) allowed for a rapid lysis of tumor cells followed by a secondary tumor-specific T cell-mediated antitumor immune response [239]. Based on high rates of complete response and durable local control achieved in metastatic cutaneous melanoma, PV-10 chemoablation has been evaluated in a phase I safety and tolerability study in mUM [240].

### 6.4. Targeted Therapy 

Recent advances in the molecular profiling of UM provide a rationale for treatments that selectively target the effectors of the molecular pathways which regulate tumor growth. Specifically, mutations of the GNAQ and GNA11 genes encoding for Gα subunits of G-proteins drive oncogenesis in most of primary and mUM, whereas mutations in the phospholipase C4 (PLCB4) or in the Cysteinyl Leukotriene Receptor 2 (CYSLTR2) genes occur less frequently [241,242]. The development of therapies aimed at directly targeting Gα proteins is still in an initial phase in mUM, whereas BRAF (v-raf murine sarcoma viral oncogene homolog B1)-targeted therapy has achieved substantial results in cutaneous melanoma [243,244]. The cyclic depsipeptide FR900359 (FR) was observed to allosterically inhibit the GDP/GTP exchange to obtain inactive Gαβγ heterotrimers from constitutively active GαQ and 11, thus promoting cell cycle arrest in UM cells in culture and inhibiting tumor growth in UM mouse xenografts [245,246]. The design of simplified analogues of FR900359 capable of effective GαQ/11 inhibition, including the small molecule YM-19, opens new perspectives for pharmaceutical development [247]. Among gene regulatory approaches, a combination therapy of oncolytic adenovirus H101 and siRNA mediating GNAQ downregulation was shown to induce UM cells apoptosis in in vivo activating UM cell apoptosis [248]. Moreover, a system for conjugating siRNAs to functionalized gold nanoparticles (AuNPs) able to recognize transcripts of mutant GNAQ mRNA was developed. This approach resulted in greater intracellular release of siRNA and decreased cancer cell viability [249]. GNAQ/GNA11 mutations drive the constitutive activation of the mitogen-activated protein kinase (MAPK) pathway, and therapies targeting downstream effectors of Gα at the level of MEK, PKC, and AKT have been investigated. In the phase III clinical trial SUMIT, naïve mUM patients were randomized to receive either selumetinib—a selective MEK inhibitor—or placebo, in combination with dacarbazine. Results of this study did not show a difference in the primary endpoint of PFS between the two groups of treatment [250]. The combination of selumetinib with the AKT inhibitor MK2206 resulted in synergistic suppression of GNAQ mutant cell viability in vitro and in xenograft mouse models of UM [251]. Based on these encouraging preclinical results, a randomized phase II clinical trial was performed to investigate the efficacy of trametinib—a selective MEK inhibitor—with or without AKT inhibition. However, this study did not demonstrate any substantial improvement in the primary endpoint of response (PFS) for combinational treatment [252]. Sorafenib—a kinase inhibitor targeting RAF/MEK/ERK pathway and VEGFR/PDGFR—was investigated in a phase II study by the Southwest Oncology Group (SWOG) cooperative group in combination with carboplatin and paclitaxel in mUM, but the limited overall efficacy did not warrant further clinical tests [253]. Among other strategies, a phase I study is ongoing evaluating the preliminary antitumor activity of LXS196, a PKC inhibitor, as monotherapy and in combination with HDM201 (MDM2 inhibitor) in patients with mUM [254]. A number of phase I and II trials on other targeted therapies in mUM have been completed, demonstrating no impact on survival indicators, including lenalidomide (TNF-α secretion inhibitor), gefitinib (epidermal growth factor inhibitor), bevacizumab and aflibercept (vascular endothelial growth factor inhibitors), imatinib (KIT inhibitor), sunitinib (tyrosine kinase inhibitor), vorinostat (histone deacetylase inhibitor), carbozantinib (tyrosine kinases c-Met and VEGFR2 inhibitor), cixutumumab (IGF1R inhibitor), everoliumus (mTOR inhibitor) plus pasireotide (a somatostatin analog), and ganetespib (heat-shock protein 90 inhibitor) ([196,255,256,257,258,259,260,261,262,263,264,265,266,267,268,269]. Currently active phase I/II trials in mUM, targeting molecules other than MEK, AKT, and PKC, are based on BVD-523 (ERK1/ERK2 inhibitor), BPX-701 (a genetically modified autologous T cell product incorporating an HLA-A2-restricted PRAME-directed TCR), and cabozantinib (multikinase inhibitor) versus temozolomide or dacarbazine [270,271,272]. The main trials currently evaluating liver-directed therapies, immunotherapies, and targeted therapies for mUM are listed in Table 3.

## 7. Conclusions

UM represents a challenge for oncologists and ophthalmologists in terms of early diagnosis, clinical and genetic characterization, and treatments. In recent decades, considerable advances have been made in the diagnosis and classification of patients at low/high-risk of metastatic progression in UM, thereby facilitating early and tailored intervention. However, 50% of patients still develop metastatic disease, and survival rates do not show substantial improvements. Thus far, there is no accepted standard of care for the treatment of UM in adjuvant and metastatic settings, and most of current treatments for UM are adapted from results observed in cutaneous melanoma, although UM shows different clinical and molecular features from its cutaneous counterpart. The increasing knowledge of tumor biology, genetics, and immunology has recently led to UM-specific clinical trials for adjuvant and metastatic scope. Specifically, insights into the primary and metastatic UM microenvironment and tumor immune surveillance, as well as the mechanisms regulating metastatic tumor dormancy, paved the way for the identification of targets for future therapies. Therefore, research should be focused on testing novel promising therapies, and continued participation in clinical trials should be encouraged. This will hopefully increase the survival benefit of UM patients similarly to what has recently been observed for cutaneous melanoma. 

## Figures and Tables

**Table 1:** Features predicting malignant transformation of choroidal nevus into melanoma are listed in the mnemonic ”TFSOM UHHD ‘to find small ocular melanoma using helpful hints daily’”. The percentages of choroidal nevus growth into melanoma based on number of involved features are reported along with recommended clinical monitoring [20,21].

Mnemonic	Feature	N of Features	Choroidal Nevus Growth into Melanoma (%)	Monitoring
To Find Small Ocular Melanoma Using helpful Hints Daily	Thickness > 2 mm	None	4%	Every 6 months 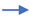 Once a year (if stability persists)
	Fluid (subretinal)			
	Symptoms - decreased vision - flashes/floaters	1 Feature	36%	Every 4–6 months
	Orange pigment			
	Margin ≤ 3 mm to disc	2 Features	45%	Every 4–6 months
	Ultrasonographic hollowness			
	Halo absence Drusen absence	3 or more Features	50%	Referral to Experienced Center 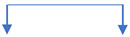 Primary treatment Prognosis

**Table 2:** Current adjuvant trials in uveal melanoma.

Clinical Trials N	Tested Agent and Mechanism of Action	Phase	Status
NCT02223819	Crizotinib (c-Met inhibitor)	II	Recruiting
NCT02068586	Sunitinib (c-Kit inhibitor) vs. Valproic acid (HDAC inhibitor)	II	Recruiting
NCT00489944	Suntinib (c-Kit inhibitor) + Tamoxifen (estrogen receptor modulator) + Cisplatin (alkylating agent)	II	Unknown
NCT01983748	Dendritic cell vaccination (immunotherapy)	III	Recruiting
NCT00929019	Dendritic cell vaccination (immunotherapy)	I/II	Terminated, slow accrual
NCT02519322	Nivolumab (anti-PD1) with or without Ipilimumab (anti-CTLA4) or Relatlimab (anti-LAG3)	II	Recruiting
NCT03528408	Ipilimumab (anti-CTLA4) + Nivolumab(anti-PD1)	II	Recruiting
NCT02336763	Prophylactic External-Beam Radiation Therapy to the liver	II	Terminated, lack of accrual

**Table 3:** Main ongoing trials for metastatic uveal melanoma.

Clinical Trials N	Tested Agent and Mechanism of Action	Phase	Status
NCT01785316	IHP with melphalan or best alternative care	III	Recruiting
NCT02678572	PHP with melphalan or best alternative care	III	Recruiting
NCT01893099	^90^Y-labelled microspheres and sorafenib (inhibitor of RAF/MEK/ERK and VEGFR/PDGFR)	I	Complete, no results
NCT02626962	Nivolumab (anti-PD1) + ipilimumab (anti-CTLA4)	II	Active, not recruiting
NCT01585194	Nivolumab (anti-PD1) + ipilimumab (anti-CTLA4)	II	Active, not recruiting
NCT02697630	Pembrolizumab (anti-PD1) + Entinostat (HDAC inhibitor)	II	Active, not recruiting
NCT04283890	PHP with melphalan + ipilimumab (anti-CTLA4) and nivolumab (anti-PD1)	I/II	Recruiting
NCT02913417	^90^Y-labelled microspheres + Ipilimumab (anti-CTLA4) and nivolumab (anti-PD1)	I/II	Recruiting
NCT03472586	Immunoembolization + Ipilimumab (anti-CTLA4) and nivolumab (anti-PD1)	II	Recruiting
NCT03467516	TILs	II	Recruiting
NCT03068624	TILs + cyclophosphamide (alkylating agent), aldesleukin (human recombinant IL-2), and ipilimumab (anti-CTLA4)	Ib	Active, not recruiting
NCT02570308	ImmTAC molecule (IMCgp100) targeting gp100	I/II	Active, not recruiting
NCT03070392	ImmTAC molecule (IMCgp100) targeting gp100Vs. investigator’s choice	II	Active, not recruiting.
NCT03635632	C7R-GD2.CAR T cells	I	Recruiting
NCT00219843	Intralesional (IL) PV-10 chemoablation(rose bengal disodium, 10%)	I	Complete, no results
NCT01979523	Trametinib (MEK inhibitor) ± AKT inhibition	II	Complete, has results
NCT02601378	LXS196 (PKC inhibitor) ± HDM201 (MDM2 inhibitor)	I	Active, not recruiting
NCT01413191	Cixutumumab (IGF1R inhibitor)	II	Complete, has results
NCT01252251	Everoliumus (mTOR inhibitor) and pasireotide (somatostatin analog)	II	Complete, has results
NCT03417739	BVD-523(ERK1/ERK2 inhibitor)	II	Active, not recruiting
NCT02743611	BPX-701 (PRAME-targeting T-cell receptor)	I/II	Active, not recruiting
NCT01835145	Carbozantinib (c-MET, c-KIT, VEGFR2 inhibitor) vs. temozolomide (alkylating agent) or dacarbazine (alkylating agent)	II	Active, not recruiting, has results

Abbreviations: Isolated hepatic perfusion (IHP), Percutaneous hepatic perfusion (PHP), immunoembolization (IE), tumor-infiltrating lymphocytes (TILs), chimeric antigen receptors (CAR), Immune-mobilizing monoclonal TCRs against cancer (ImmTAC).

## References

[1] Chang A.E., Karnell L.H., Menck H.R. (1998). The national cancer data base report on cutaneous and noncutaneous melanoma: A summary of 84,836 cases from the past decade. Cancer.

[2] Shields C.L., Kaliki S., Furuta M., Mashayekhi A., Shields J.A. (2012). Clinical spectrum and prognosis of uveal melanoma based on age at presentation in 8033 cases. Retina.

[3] McLaughlin C.C., Wu X.C., Jemal A., Martin H.J., Roche L.M., Chen V.W. (2005). Incidence of noncutaneous melanomas in the U.S. Cancer.

[4] Singh A.D., Turell M.E., Topham A.K. (2011). Uveal melanoma: Trends in incidence, treatment, and survival. Ophthalmology.

[5] Virgili G., Gatta G., Ciccolallo L., Capocaccia R., Biggeri A., Crocetti E., Lutz J.M., Paci E. (2007). Incidence of Uveal Melanoma in Europe. Ophthalmology.

[6] Mallone S., De Vries E., Guzzo M., Midena E., Verne J., Coebergh J.W., Marcos-Gragera R., Ardanaz E., Martinez R., Chirlaque M.D. (2012). Descriptive epidemiology of malignant mucosal and uveal melanomas and adnexal skin carcinomas in Europe. Eur. J. Cancer.

[7] Damato E.M., Damato B.E. (2012). Detection and time to treatment of uveal melanoma in the United Kingdom: An evaluation of 2384 patients. Ophthalmology.

[8] Mahendraraj K., Lau C.S.M., Lee I., Chamberlain R.S. (2016). Trends in incidence, survival, and management of uveal melanoma: A population-based study of 7,516 patients from the surveillance, epidemiology, and end results database (1973–2012). Clin. Ophthalmol..

[9] Aronow M.E., Topham A.K., Singh A.D. (2018). Uveal Melanoma: 5-Year Update on Incidence, Treatment, and Survival (SEER 1973-2013). Ocul. Oncol. Pathol..

[10] Xu Y., Lou L., Wang Y., Miao Q., Jin K., Chen M., Ye J. (2020). Epidemiological Study of Uveal Melanoma from US Surveillance, Epidemiology, and End Results Program (2010–2015). J. Ophthalmol..

[11] Hu D.N., Yu G.P., McCormick S.A., Schneider S., Finger P.T. (2005). Population-based incidence of uveal melanoma in various races and ethnic groups. Am. J. Ophthalmol..

[12] Yu G.-P., Hu D.-N., McCormick S.A. (2006). Latitude and Incidence of Ocular Melanoma. Photochem. Photobiol..

[13] Shields C.L., Kaliki S., Cohen M.N., Shields P.W., Furuta M., Shields J.A. (2015). Prognosis of uveal melanoma based on race in 8100 patients: The 2015 Doyne Lecture. Eye.

[14] Shields C.L., Kaliki S., Livesey M., Walker B., Garoon R., Bucci M., Feinstein E., Pesch A., Gonzalez C., Lally S.E. (2013). Association of ocular and oculodermal melanocytosis with the rate of uveal melanoma metastasis analysis of 7872 consecutive eyes. JAMA Ophthalmol..

[15] Singh A.D., De Potter P., Fijal B.A., Shields C.L., Shields J.A., Elston R.C. (1998). Lifetime prevalence of uveal melanoma in white patients with oculo(dermal) melanocytosis. Ophthalmology.

[16] Hammer H., Oláh J., Tóth-Molnár E. (1996). Dysplastic nevi are a risk factor for uveal melanoma. Eur. J. Ophthalmol..

[17] McDonald K.A., Krema H., Chan A.-W. (2020). Cutaneous Signs and Risk Factors for Ocular Melanoma. J. Am. Acad. Dermatol..

[18] Barker C.A., Salama A.K. (2018). New NCCN guidelines for uveal melanoma and treatment of recurrent or progressive distant metastatic melanoma. J. Natl. Compr. Cancer Netw..

[19] Rodrigues M., de Koning L., Coupland S.E., Jochemsen A.G., Marais R., Stern M.H., Valente A., Barnhill R., Cassoux N., Evans A. (2019). So close, yet so far: Discrepancies between uveal and other melanomas. a position paper from UM cure 2020. Cancers.

[20] Shields C.L., Cater J., Shields J.A., Singh A.D., Santos M.C.M., Carvalho C. (2000). Combination of clinical factors predictive of growth of small choroidal melanocytic tumors. Arch. Ophthalmol..

[21] Shields C.L., Furuta M., Berman E.L., Zahler J.D., Hoberman D.M., Dinh D.H., Mashayekhi A., Shields J.A. (2009). Choroidal nevus transformation into melanoma: Analysis of 2514 consecutive cases. Arch. Ophthalmol..

[22] Melia B.M., Diener-West M., Bennett S.R., Folk J.C., Montague P.R., Weingeist T.A., Hawkins B.S. (1997). Factors predictive of growth and treatment of small choroidal melanoma: COMS report no. 5. Arch. Ophthalmol..

[23] (1990). Accuracy of Diagnosis of Choroidal Melanomas in the Collaborative Ocular Melanoma Study: COMS Report No. 1. Arch. Ophthalmol..

[24] Shields J.A., Shields C.L., Ehya H., Eagle R.C., De Potter P. (1993). Fine-needle aspiration biopsy of suspected intraocular tumors. Int. Ophthalmol. Clin..

[25] Hawkins B.S. (2006). The COMS randomized trial of iodine 125 brachytherapy for choroidal melanoma: V. Twelve-year mortality rates and prognostic factors: COMS report no. 28. Arch. Ophthalmol..

[26] Kujala E., Mäkitie T., Kivelä T. (2003). Very Long-Term Prognosis of Patients with Malignant Uveal Melanoma. Investig. Ophthalmol. Vis. Sci..

[27] Hawkins B.S. (2004). The Collaborative Ocular Melanoma Study (COMS) randomized trial of pre-enucleation radiation of large choroidal melanoma: IV. Ten-year mortality findings and prognostic factors. COMS report number 24. Am. J. Ophthalmol..

[28] Bishop K.D., Olszewski A.J. (2014). Epidemiology and survival outcomes of ocular and mucosal melanomas: A population-based analysis. Int. J. Cancer.

[29] Burr J.M., Mitry E., Rachet B., Coleman M.P. (2007). Survival from uveal melanoma in England and Wales 1986 to 2001. Ophthalmic Epidemiol..

[30] Kuk D., Shoushtari A.N., Barker C.A., Panageas K.S., Munhoz R.R., Momtaz P., Ariyan C.E., Brady M.S., Coit D.G., Bogatch K. (2016). Prognosis of Mucosal, Uveal, Acral, Nonacral Cutaneous, and Unknown Primary Melanoma From the Time of First Metastasis. Oncologist.

[31] Seibel I., Cordini D., Rehak M., Hager A., Riechardt A.I., Böker A., Heufelder J., Weber A., Gollrad J., Besserer A. (2015). Local Recurrence after Primary Proton Beam Therapy in Uveal Melanoma: Risk Factors, Retreatment Approaches, and Outcome. Am. J. Ophthalmol..

[32] Coupland S.E., Sidiki S., Clark B.J., McClaren K., Kyle P., Lee W.R. (1996). Metastatic choroidal melanoma to the contralateral orbit 40 years after enucleation. Arch. Ophthalmol..

[33] Shields J.A., Augsburger J.J., Donoso L.A., Bernardino V.B., Portenar M. (1985). Hepatic metastasis and orbital recurrence of uveal melanoma after 42 years. Am. J. Ophthalmol..

[34] Dithmar S., Diaz C.E., Grossniklaus H.E. (2000). Intraocular melanoma spread to regional lymph nodes. Retina.

[35] Badve S.S., Fisher C. (2020). AJCC 8th edition—A step forward. Breast J..

[36] Shields C.L., Furuta M., Thangappan A., Nagori S., Mashayekhi A., Lally D.R., Kelly C.C., Rudich D.S., Nagori A.V., Wakade O.A. (2009). Metastasis of uveal melanoma millimeter-by-millimeter in 8033 consecutive eyes. Arch. Ophthalmol..

[37] Shields C.L., Kaliki S., Furuta M., Fulco E., Alarcon C., Shields J.A. (2013). American Joint Committee on Cancer classification of posterior uveal melanoma (tumor size category) predicts prognosis in 7731 patients. Ophthalmology.

[38] Gill H.S., Char D.H. (2012). Uveal melanoma prognostication: From lesion size and cell type to molecular class. Can. J. Ophthalmol..

[39] Bagger M., Andersen M.T., Andersen K.K., Heegaard S., Kiilgaard J.F. (2015). The prognostic effect of American joint committee on cancer staging and genetic status in patients with choroidal and ciliary body melanoma. Investig. Ophthalmol. Vis. Sci..

[40] Amin M.B., Greene F.L., Edge S.B., Compton C.C., Gershenwald J.E., Brookland R.K., Meyer L., Gress D.M., Byrd D.R., Winchester D.P. (2017). The Eighth Edition AJCC Cancer Staging Manual: Continuing to build a bridge from a population-based to a more “personalized” approach to cancer staging. CA Cancer J. Clin..

[41] Cassoux N., Rodrigues M.J., Plancher C., Asselain B., Levy-Gabriel C., Lumbroso-Le Rouic L., Piperno-Neumann S., Dendale R., Sastre X., Desjardins L. (2014). Genome-wide profiling is a clinically relevant and affordable prognostic test in posterior uveal melanoma. Br. J. Ophthalmol..

[42] Damato B., Duke C., Coupland S.E., Hiscott P., Smith P.A., Campbell I., Douglas A., Howard P. (2007). Cytogenetics of Uveal Melanoma. A 7-Year Clinical Experience. Ophthalmology.

[43] Shields C.L., Say E.A.T., Hasanreisoglu M., Saktanasate J., Lawson B.M., Landy J.E., Badami A.U., Sivalingam M.D., Hauschild A.J., House R.J. (2017). Personalized Prognosis of Uveal Melanoma Based on Cytogenetic Profile in 1059 Patients over an 8-Year Period: The 2017 Harry S. Gradle Lecture. Ophthalmology.

[44] Onken M.D., Worley L.A., Ehlers J.P., Harbour J.W. (2004). Gene expression profiling in uveal melanoma reveals two molecular classes and predicts metastatic death. Cancer Res..

[45] Onken M.D., Worley L.A., Char D.H., Augsburger J.J., Correa Z.M., Nudleman E., Aaberg T.M., Altaweel M.M., Bardenstein D.S., Finger P.T. (2012). Collaborative ocular oncology group report number 1: Prospective validation of a multi-gene prognostic assay in uveal melanoma. Ophthalmology.

[46] Damato B., Coupland S.E. (2009). A reappraisal of the significance of largest basal diameter of posterior uveal melanoma. Eye.

[47] Damato B., Dopierala J., Klaasen A., van Dijk M., Sibbring J., Coupland S.E. (2009). Multiplex ligation-dependent probe amplification of uveal melanoma: Correlation with metastatic death. Investig. Ophthalmol. Vis. Sci..

[48] Vaquero-Garcia J., Lalonde E., Ewens K.G., Ebrahimzadeh J., Richard-Yutz J., Shields C.L., Barrera A., Green C.J., Barash Y., Ganguly A. (2017). PRiMeUM: A model for predicting risk of metastasis in uveal melanoma. Investig. Ophthalmol. Vis. Sci..

[49] Walter S.D., Chao D.L., Feuer W., Schiffman J., Char D.H., Harbour J.W. (2016). Prognostic implications of tumor diameter in association with gene expression profile for uveal melanoma. JAMA Ophthalmol..

[50] Eleuteri A., Damato B., Coupland S.E., Taktak A.F.G. (2012). Enhancing survival prognostication in patients with choroidal melanoma by integrating pathologic clinical and genetic predictors of metastasis. Int. J. Biomed. Eng. Technol..

[51] Jager M.J., Brouwer N.J., Esmaeli B. (2018). The Cancer Genome Atlas Project: An Integrated Molecular View of Uveal Melanoma. Ophthalmology.

[52] Mazloumi M., Vichitvejpaisal P., Dalvin L.A., Yaghy A., Ewens K.G., Ganguly A., Shields C.L. (2020). Accuracy of the Cancer Genome Atlas Classification vs American Joint Committee on Cancer Classification for Prediction of Metastasis in Patients with Uveal Melanoma. JAMA Ophthalmol..

[53] Robertson A.G., Shih J., Yau C., Gibb E.A., Oba J., Mungall K.L., Hess J.M., Uzunangelov V., Walter V., Danilova L. (2017). Integrative Analysis Identifies Four Molecular and Clinical Subsets in Uveal Melanoma. Cancer Cell.

[54] Vichitvejpaisal P., Dalvin L.A., Mazloumi M., Ewens K.G., Ganguly A., Shields C.L. (2019). Genetic Analysis of Uveal Melanoma in 658 Patients Using the Cancer Genome Atlas Classification of Uveal Melanoma as A, B, C, and D. Ophthalmology.

[55] Harbour J.W. (2012). The genetics of uveal melanoma: An emerging framework for targeted therapy. Pigment Cell Melanoma Res..

[56] Prescher G., Bornfeld N., Hirche H., Horsthemke B., Jöckel K.H., Becher R. (1996). Prognostic implications of monosomy 3 in uveal melanoma. Lancet.

[57] Tschentscher F., Prescher G., Zeschnigk M., Horsthemke B., Lohmann D.R. (2000). Identification of chromosomes 3, 6, and 8 aberrations in uveal melanoma by microsatellite analysis in comparison to comparative genomic hybridization. Cancer Genet. Cytogenet..

[58] Versluis M., De Lange M.J., Van Pelt S.I., Ruivenkamp C.A.L., Kroes W.G.M., Cao J., Jager M.J., Luyten G.P.M., Van Der Velden P.A. (2015). Digital PCR validates 8q dosage as prognostic tool in uveal melanoma. PLoS ONE.

[59] Harbour J.W., Onken M.D., Roberson E.D.O., Duan S., Cao L., Worley L.A., Council M.L., Matatall K.A., Helms C., Bowcock A.M. (2010). Frequent mutation of BAP1 in metastasizing uveal melanomas. Science.

[60] Carbone M., Ferris L.K., Baumann F., Napolitano A., Lum C.A., Flores E.G., Gaudino G., Powers A., Bryant-Greenwood P., Krausz T. (2012). BAP1 cancer syndrome: Malignant mesothelioma, uveal and cutaneous melanoma, and MBAITs. J. Transl. Med..

[61] Laíns I., Bartosch C., Mondim V., Healy B., Kim I.K., Husain D., Miller J.W. (2016). Second Primary Neoplasms in Patients With Uveal Melanoma: A SEER Database Analysis. Am. J. Ophthalmol..

[62] Figueiredo C.R., Kalirai H., Sacco J.J., Azevedo R.A., Duckworth A., Slupsky J.R., Coulson J.M., Coupland S.E. (2020). Loss of BAP1 expression is associated with an immunosuppressive microenvironment in uveal melanoma, with implications for immunotherapy development. J. Pathol..

[63] Karlsson J., Nilsson L.M., Mitra S., Alsén S., Shelke G.V., Sah V.R., Forsberg E.M.V., Stierner U., All-Eriksson C., Einarsdottir B. (2020). Molecular profiling of driver events in metastatic uveal melanoma. Nat. Commun..

[64] Louie B.H., Kurzrock R. (2020). BAP1: Not Just a BRCA1-Associated Protein. Cancer Treat. Rev..

[65] Yavuzyigitoglu S., Koopmans A.E., Verdijk R.M., Vaarwater J., Eussen B., Van Bodegom A., Paridaens D., Kiliç E., De Klein A. (2016). Uveal Melanomas with SF3B1 Mutations: A Distinct Subclass Associated with Late-Onset Metastases. Ophthalmology.

[66] Harbour J.W., Chen R. (2013). The DecisionDx-UM Gene Expression Profile Test Provides Risk Stratification and Individualized Patient Care in Uveal Melanoma. PLoS Curr..

[67] Luscan A., Just P.A., Briand A., Burin Des Roziers C., Goussard P., Nitschké P., Vidaud M., Avril M.F., Terris B., Pasmant E. (2015). Uveal melanoma hepatic metastases mutation spectrum analysis using targeted next-generation sequencing of 400 cancer genes. Br. J. Ophthalmol..

[68] Martin M., Maßhöfer L., Temming P., Rahmann S., Metz C., Bornfeld N., Van De Nes J., Hitpass L.K., Hinnebusch A.G., Horsthemke B. (2013). Exome sequencing identifies recurrent somatic mutations in EIF1AX and SF3B1 in uveal melanoma with disomy 3. Nat. Genet..

[69] Höglund M., Gisselsson D., Hansen G.B., White V.A., Säll T., Mitelman F., Horsman D. (2004). Dissecting karyotypic patterns in malignant melanomas: Temporal clustering of losses and gains in melanoma karyotypic evolution. Int. J. Cancer.

[70] Damato B.E., Heimann H., Kalirai H., Coupland S.E. (2014). Age, survival predictors, and metastatic death in patients with choroidal melanoma tentative evidence of a therapeutic effect on survival. JAMA Ophthalmol..

[71] Aalto Y., Eriksson L., Seregard S., Larsson O., Knuutila S. (2001). Concomitant loss of chromosome 3 and whole arm losses and gains of chromosome 1, 6, or 8 in metastasizing primary uveal melanoma. Investig. Ophthalmol. Vis. Sci..

[72] Ewens K.G., Kanetsky P.A., Richards-Yutz J., Al-Dahmash S., de Luca M.C., Bianciotto C.G., Shields C.L., Ganguly A. (2013). Genomic profile of 320 uveal melanoma cases: Chromosome 8p-loss and metastatic outcome. Investig. Ophthalmol. Vis. Sci..

[73] Naus N.C., Verhoeven A.C.A., van Drunen E., Slater R., Mooy C.M., Paridaens D.A., Luyten G.P.M., de Klein A. (2002). Detection of Genetic Prognostic Markers in Uveal Melanoma Biopsies Using Fluorescence in Situ Hybridization. Clin. Cancer Res..

[74] Sisley K., Nichols C., Parsons M.A., Farr R., Rees R.C., Rennie I.G. (1998). Clinical applications of chromosome analysis, from fine needle aspiration biopsies, of posterior uveal melanomas. Eye.

[75] Shields C.L., Ganguly A., Materin M.A., Teixeira L., Mashayekhi A., Swanson L.A., Marr B.P., Shields J.A. (2007). Chromosome 3 analysis of uveal melanoma using fine-needle aspiration biopsy at the time of plaque radiotherapy in 140 consecutive cases: The Deborah Iverson, MD, Lectureship. Arch. Ophthalmol..

[76] Klofas L.K., Bogan C.M., Coogan A., Schultenover S.J., Weiss V.L., Daniels A.B. (2020). Instrument gauge and type in uveal melanoma fine needle biopsy: Implications for diagnostic yield and molecular prognostication. Am. J. Ophthalmol..

[77] Young T.A., Rao N.P., Glasgow B.J., Moral J.N., Straatsma B.R. (2007). Fluorescent In Situ Hybridization for Monosomy 3 via 30-Gauge Fine-Needle Aspiration Biopsy of Choroidal Melanoma In Vivo. Ophthalmology.

[78] Cross N.A., Ganesh A., Parpia M., Murray A.K., Rennie I.G., Sisley K. (2006). Multiple locations on chromosome 3 are the targets of specific deletions in uveal melanoma. Eye.

[79] Lake S.L., Kalirai H., Dopierała J., Damato B.E., Coupland S.E. (2012). Comparison of Formalin-Fixed and Snap-Frozen Samples Analyzed by Multiplex Ligation-Dependent Probe Amplification for Prognostic Testing in Uveal Melanoma. Investig. Ophthalmol. Vis. Sci..

[80] Worley L.A., Onken M.D., Person E., Robirds D., Branson J., Char D.H., Perry A., Harbour J.W. (2007). Transcriptomic versus chromosomal prognostic markers and clinical outcome in uveal melanoma. Clin. Cancer Res..

[81] Onken M.D., Worley L.A., Tuscan M.D., Harbour J.W. (2010). An accurate, clinically feasible multi-gene expression assay for predicting metastasis in uveal melanoma. J. Mol. Diagn..

[82] Field M.G., Harbour J.W. (2014). Recent developments in prognostic and predictive testing in uveal melanoma. Curr. Opin. Ophthalmol..

[83] Field M.G., Decatur C.L., Kurtenbach S., Gezgin G., Van Der Velden P.A., Jager M.J., Kozak K.N., Harbour J.W. (2016). PRAME as an independent biomarker for metastasis in uveal melanoma. Clin. Cancer Res..

[84] Triozzi P.L., Singh A.D. (2014). Adjuvant Therapy of Uveal Melanoma: Current Status. Ocul. Oncol. Pathol..

[85] Seth R., Messersmith H., Kaur V., Kirkwood J.M., Kudchadkar R., McQuade J.L., Provenzano A., Swami U., Weber J., Alluri K.C. (2020). Systemic Therapy for Melanoma: ASCO Guideline. J. Clin. Oncol..

[86] Marshall E., Romaniuk C., Ghaneh P., Wong H., McKay M., Chopra M., Coupland S.E., Damato B.E. (2013). MRI in the detection of hepatic metastases from high-risk uveal melanoma: A prospective study in 188 patients. Br. J. Ophthalmol..

[87] Nathan P., Cohen V., Coupland S., Curtis K., Damato B., Evans J., Fenwick S., Kirkpatrick L., Li O., Marshall E. (2015). Uveal Melanoma UK National Guidelines. Eur. J. Cancer.

[88] Desjardins L., Dorval T., Levy C., Cojean I., Schlienger P., Salmon R.J., Validire P., Asselain B. (1998). Etude randomisée de chimiothérapie adjuvante par le Déticène dans le mélanome choroïdien. Ophtalmologie.

[89] McLean I.W., Berd D., Mastrangelo M.J., Shields J.A., Davidorf F.H., Grever M., Makley T.A., Gamel J.W. (1990). A randomized study of methanol-extraction residue of bacille Calmette-Guerin as postsurgical adjuvant therapy of uveal melanoma. Am. J. Ophthalmol..

[90] Richtig E., Langmann G., Schlemmer G., Müllner K., Papaefthymiou G., Bergthaler P., Smolle J. (2006). Verträglichkeit und wirksamkeit einer adjuvanten interferon-alfa-2b-behandlung beim aderhautmelanom. Ophthalmologe.

[91] Lane A.M., Egan K.M., Harmon D., Holbrook A., Munzenrider J.E., Gragoudas E.S. (2009). Adjuvant Interferon Therapy for Patients with Uveal Melanoma at High Risk of Metastasis. Ophthalmology.

[92] Voelter V., Schalenbourg A., Pampallona S., Peters S., Halkic N., Denys A., Goitein G., Zografos L., Leyvraz S. (2008). Adjuvant intra-arterial hepatic fotemustine for high-risk uveal melanoma patients. Melanoma Res..

[93] Piperno-Neumann S., Rodrigues M.J., Servois V., Pierron G., Gastaud L., Negrier S., Levy-Gabriel C., Lumbroso L., Cassoux N., Bidard F.-C. (2017). A randomized multicenter phase 3 trial of adjuvant fotemustine versus surveillance in high risk uveal melanoma (UM) patients (FOTEADJ). J. Clin. Oncol..

[94] Binkley E., Triozzi P.L., Rybicki L., Achberger S., Aldrich W., Singh A. (2020). A prospective trial of adjuvant therapy for high-risk uveal melanoma: Assessing 5-year survival outcomes. Br. J. Ophthalmol..

[95] Sato T., Han F., Yamamoto A. (2008). The biology and management of uveal melanoma. Curr. Oncol. Rep..

[96] Surriga O., Rajasekhar V.K., Ambrosini G., Dogan Y., Huang R., Schwartz G.K. (2013). Crizotinib, a c-Met Inhibitor, Prevents Metastasis in a Metastatic Uveal Melanoma Model. Mol. Cancer Ther..

[97] Search of: NCT02223819—List Results—ClinicalTrials.gov. NCT02223819.

[98] Valsecchi M.E., Orloff M., Sato R., Chervoneva I., Shields C.L., Shields J.A., Mastrangelo M.J., Sato T. (2018). Adjuvant Sunitinib in High-Risk Patients with Uveal Melanoma: Comparison with Institutional Controls. Ophthalmology.

[99] Search of: NCT00489944—List Results—ClinicalTrials.gov. NCT00489944.

[100] Search of: NCT02068586—List Results—ClinicalTrials.gov. NCT02068586.

[101] Landreville S., Agapova O.A., Matatall K.A., Kneass Z.T., Onken M.D., Lee R.S., Bowcock A.M., Harbour J.W. (2011). Histone Deacetylase Inhibitors Induce Growth Arrest and Differentiation in Uveal Melanoma. AACR.

[102] Fagone P., Caltabiano R., Russo A., Lupo G., Anfuso C.D., Basile M.S., Longo A., Nicoletti F., De Pasquale R., Libra M. (2017). Identification of novel chemotherapeutic strategies for metastatic uveal melanoma. Sci. Rep..

[103] Bol K., van den Bosch T., Schreibelt G., Punt C., Figdor C., Paridaens D., de Vries J. (2015). Adjuvant dendritic cell vaccination in high-risk uveal melanoma patients. J. Immunother. Cancer.

[104] Search of: NCT01983748—List Results—ClinicalTrials.gov. NCT01983748.

[105] Search of: NCT00929019—List Results—ClinicalTrials.gov. NCT00929019.

[106] Schank T.E., Hassel J.C. (2019). cancers Immunotherapies for the Treatment of Uveal Melanoma-History and Future. Cancers.

[107] Durante M.A., Rodriguez D.A., Kurtenbach S., Kuznetsov J.N., Sanchez M.I., Decatur C.L., Snyder H., Feun L.G., Livingstone A.S., Harbour J.W. (2020). Single-cell analysis reveals new evolutionary complexity in uveal melanoma. Nat. Commun..

[108] Fountain E., Bassett R.L., Cain S., Posada L., Gombos D.S., Hwu P., Bedikian A., Patel S.P. (2019). Adjuvant ipilimumab in high-risk Uveal melanoma. Cancers.

[109] Search of: NCT02519322—List Results—ClinicalTrials.gov. NCT02519322.

[110] Search of: NCT03528408—List Results—ClinicalTrials.gov. NCT03528408.

[111] Search of: NCT02336763—List Results—ClinicalTrials.gov. NCT02336763.

[112] Bustamante P., Miyamoto D., Goyeneche A., de Alba Graue P.G., Jin E., Tsering T., Dias A.B., Burnier M.N., Burnier J.V. (2019). Beta-blockers exert potent anti-tumor effects in cutaneous and uveal melanoma. Cancer Med..

[113] Croock D.L. (2002). Metastatic uveal melanoma: Diagnosis and treatment. A literature review. Bull. Société Belg. Ophtalmol..

[114] Freton A., Chin K.J., Raut R., Tena L.B., Kivelä T., Finger P.T. (2011). Initial PET/CT staging for choroidal melanoma: AJCC correlation and second nonocular primaries in 333 patients. Eur. J. Ophthalmol..

[115] Grossniklaus H.E. (2019). Understanding Uveal Melanoma Metastasis to the Liver: The Zimmerman Effect and the Zimmerman Hypothesis. Ophthalmology.

[116] Singh A.D. (2001). Uveal melanoma: Implications of tumor doubling time. Ophthalmology.

[117] Torres V., Triozzi P., Eng C., Tubbs R., Schoenfiled L., Crabb J.W., Saunthararajah Y., Singh A.D. (2011). Circulating tumor cells in uveal melanoma. Futur. Oncol..

[118] Ossowski L., Aguirre-Ghiso J.A. (2010). Dormancy of metastatic melanoma. Pigment Cell Melanoma Res..

[119] Blanco P.L., Lim L.A., Miyamoto C., Burnier M.N. (2012). Uveal melanoma dormancy: An acceptable clinical endpoint?. Melanoma Res..

[120] Ah-Fat F.G., Damato B.E. (1998). Delays in the diagnosis of uveal melanoma and effect on treatment. Eye.

[121] Rietschel P., Panageas K.S., Hanlon C., Patel A., Abramson D.H., Chapman P.B. (2005). Variates of survival in metastatic uveal melanoma. J. Clin. Oncol..

[122] Mouriaux F., Zaniolo K., Bergeron M.A., Weidmann C., De La Fouchardière A., Fournier F., Droit A., Morcos M.W., Landreville S., Guérin S.L. (2016). Effects of long-term serial passaging on the characteristics and properties of cell lines derived from uveal melanoma primary tumors. Investig. Ophthalmol. Vis. Sci..

[123] Almog N. (2010). Molecular mechanisms underlying tumor dormancy. Cancer Lett..

[124] Hedley B.D., Allan A.L., Chambers A.F. (2006). Tumor dormancy and the role of metastasis suppressor genes in regulating ectopic growth. Futur. Oncol..

[125] Horak C.E., Lee J.H., Marshall J.C., Shreeve S.M., Steeg P.S. (2008). The role of metastasis suppressor genes in metastatic dormancy. Apmis.

[126] Vidal-Vanaclocha F. (2008). The Prometastatic Microenvironment of the Liver. Cancer Microenviron..

[127] Krishna Y., Mccarthy C., Kalirai H., Coupland S.E., Yamini K., Conni M., Helen K. (2017). Inflammatory cell infiltrates in advanced metastatic uveal melanoma. Hum. Pathol..

[128] Eyles J., Puaux A., Wang X. (2010). Tumor cells disseminate early, but immunosurveillance limits metastatic outgrowth, in a mouse model of melanoma. J. Clin. Investig..

[129] Mouriaux F., Casagrande F., Pillaire M.-J., Manenti S., Malecaze F., Darbon J.-M. (1998). Differential Expression of Gl Cyclins and Cyclin-Dependent Kinase Inhibitors in Normal and Transformed Melanocytes. Investig. Ophthalmol. Vis. Sci..

[130] Mouriaux F., Maurage C., science P.L. (2000). Cyclin-dependent kinase inhibitory protein expression in human choroidal melanoma tumors. Investig. Ophthalmol. Vis. Sci..

[131] Bronkhorst I.H.G., Jager M.J. (2012). Uveal melanoma: The inflammatory microenvironment. J. Innate Immun..

[132] Ly L., Baghat A., Versluis M., Jordanova E., Immunol G.L.-J., Luyten G.P.M., van Rooijen N., van Hall T., van der Velden P.A., Jager M.J. (2010). In aged mice, outgrowth of intraocular melanoma depends on proangiogenic M2-type macrophages. J. Immunol..

[133] Ward J., Gubin M., Schreiber R.D. (2016). The role of neoantigens in naturally occurring and therapeutically induced immune responses to cancer. Advances in Immunology.

[134] Tran E., Turcotte S., Gros A., Robbins P.F., Lu Y.C., Dudley M.E., Wunderlich J.R., Somerville R.P., Hogan K., Hinrichs C.S. (2014). Cancer immunotherapy based on mutation-specific CD4+ T cells in a patient with epithelial cancer. Science.

[135] Turcotte S., Gros A., Hogan K., Tran E., Hinrichs C.S., Wunderlich J.R., Dudley M.E., Rosenberg S.A. (2013). Phenotype and Function of T Cells Infiltrating Visceral Metastases from Gastrointestinal Cancers and Melanoma: Implications for Adoptive Cell Transfer Therapy. J. Immunol..

[136] Alexandrov L.B., Nik-Zainal S., Wedge D.C., Aparicio S.A.J.R., Behjati S., Biankin A.V., Bignell G.R., Bolli N., Borg A., Børresen-Dale A.L. (2013). Signatures of mutational processes in human cancer. Nature.

[137] Sutmuller R.P.M., Schurmans L.R.H.M., van Duivenvoorde L.M., Tine J.A., van der Voort E.I.H., Toes R.E.M., Melief C.J.M., Jager M.J., Offringa R. (2000). Adoptive T Cell Immunotherapy of Human Uveal Melanoma Targeting gp100. J. Immunol..

[138] Kittler J.M., Sommer J., Fischer A., Britting S., Karg M.M., Bock B., Atreya I., Heindl L.M., Mackensen A., Bosch J.J. (2019). Characterization of CD4+ T cells primed and boosted by MHCII primary uveal melanoma cell-based vaccines. Oncotarget.

[139] Chen W., Wang W., Chen L., Chen J., Lu X., Li Z., Wu B., Yin L., Guan Y.-Q. (2018). Long-term G 1 cell cycle arrest in cervical cancer cells induced by co-immobilized TNF-α plus IFN-γ polymeric drugs. J. Mater. Chem. B.

[140] Kortylewski M., Komyod W., Kauffmann M.E., Bosserhoff A., Heinrich P.C., Behrmann I. (2004). Interferon-γ-Mediated Growth Regulation of Melanoma Cells: Involvement of STAT1-Dependent and STAT1-Independent Signals. J. Investig. Dermatol..

[141] Schmitt M.J., Philippidou D., Reinsbach S.E., Margue C., Wienecke-Baldacchino A., Nashan D., Behrmann I., Kreis S. (2012). Interferon-γ-induced activation of Signal Transducer and Activator of Transcription 1 (STAT1) up-regulates the tumor suppressing microRNA-29 family in melanoma cells. Cell Commun. Signal..

[142] Search of: NCT01082887—List Results—ClinicalTrials.gov. NCT01082887.

[143] Huang Y., Yuan J., Righi E., Kamoun W.S., Ancukiewicz M., Nezivar J., Santosuosso M., Martin J.D., Martin M.R., Vianello F. (2012). Vascular normalizing doses of antiangiogenic treatment reprogram the immunosuppressive tumor microenvironment and enhance immunotherapy. Proc. Natl. Acad. Sci. USA.

[144] Grossniklaus H.E. (2013). Progression of ocular melanoma metastasis to the liver: The 2012 Zimmerman lecture. JAMA Ophthalmol..

[145] Grossniklaus H.E., Zhang Q., You S., McCarthy C., Heegaard S., Coupland S.E. (2016). Metastatic ocular melanoma to the liver exhibits infiltrative and nodular growth patterns. Hum. Pathol..

[146] Angi M., Kalirai H., Prendergast S., Simpson D., Hammond D.E., Madigan M.C., Beynon R.J., Coupland S.E. (2016). In-depth proteomic profiling of the uveal melanoma secretome. Oncotarget.

[147] Almog N., Ma L., Raychowdhury R., Schwager C., Erber R., Short S., Hlatky L., Vajkoczy P., Huber P.E., Folkman J. (2009). Transcriptional Switch of Dormant Tumors to Fast-Growing Angiogenic Phenotype. Cancer Res..

[148] Lee J.H., Miele M.E., Hicks D.J., Phillips K.K., Trent J.M., Weissman B.E., Welch D.R. (1996). KiSS-1, a novel human malignant melanoma metastasis-suppressor gene. J. Natl. Cancer Inst..

[149] Li J., Zhou J., Chen G., Wang H., Wang S., Xing H., Gao Q., Lu Y., He Y., Ma D. (2006). Inhibition of ovarian cancer metastasis by adeno-associated virus-mediated gene transfer of nm23H1 in an orthotopic implantation model. Cancer Gene Ther..

[150] Liu F., Qi H.-L., Chen H.-L. (2000). Effects of all-trans retinoic acid and epidermal growth factor on the expression of nm23-H1 in human hepatocarcinoma cells. J. Cancer Res. Clin. Oncol..

[151] Search of: NCT03572387—List Results—ClinicalTrials.gov. NCT03572387.

[152] Jiang Y., Berk M., Singh L.S., Tan H., Yin L., Powell C.T., Xu Y. (2005). KiSS1 suppresses metastasis in human ovarian cancer via inhibition of protein kinase C alpha. Clin. Exp. Metastasis.

[153] Takino T., Koshikawa N., Miyamori H., Tanaka M., Sasaki T., Okada Y., Seiki M., Sato H. (2003). Cleavage of metastasis suppressor gene product KiSS-1 protein/metastin by matrix metalloproteinases. Oncogene.

[154] Theodorescu D., Sapinoso L.M., Conaway M.R., Oxford G., Hampton G.M., Frierson H.F. (2004). Reduced Expression of Metastasis Suppressor RhoGDI2 Is Associated with Decreased Survival for Patients with Bladder Cancer. Clin. Cancer Res..

[155] Drake J.M., Danke J.R., Henry M.D. (2010). Bone-specific growth inhibition of prostate cancer metastasis by atrasentan. Cancer Biol. Ther..

[156] Armstrong A.J., Creel P., Turnbull J., Moore C., Jaffe T.A., Haley S., Petros W., Yenser S., Gockerman J.P., Sleep D. (2008). A Phase I-II Study of Docetaxel and Atrasentan in Men with Castration-Resistant Metastatic Prostate Cancer. Clin. Cancer Res..

[157] Quinn D.I., Tangen C.M., Hussain M., Lara P.N., Goldkorn A., Moinpour C.M., Garzotto M.G., Mack P.C., Carducci M.A., Monk J.P. (2013). Docetaxel and atrasentan versus docetaxel and placebo for men with advanced castration-resistant prostate cancer (SWOG S0421): A randomised phase 3 trial. Lancet Oncol..

[158] Witteveen P., van der Mijn K., Neoplasia M.L., Los M., Kronemeijer R.H., Groenewegen G., Voest E.E. (2010). Phase 1/2 study of atrasentan combined with pegylated liposomal doxorubicin in platinum-resistant recurrent ovarian cancer. Neoplasia.

[159] Carducci M.A., Manola J., Nair S., Liu G., Rousey S., Dutcher J.P., Wilding G. (2015). Atrasentan in Patients With Advanced Renal Cell Carcinoma: A Phase 2 Trial of the ECOG-ACRIN Cancer Research Group (E6800). Clin. Genitourin. Cancer.

[160] Santini V., Gozzini A., Ferrari G. (2007). Histone deacetylase inhibitors: Molecular and biological activity as a premise to clinical application. Current Drug Metab..

[161] Baradaran P.C., Kozovska Z., Furdova A., Smolková B. (2020). Targeting Epigenetic Modifications in Uveal Melanoma. Int. J. Mol. Sci..

[162] Van Der Kooij M.K., Speetjens F.M., Van Der Burg S.H., Kapiteijn E. (2019). Uveal Versus Cutaneous Melanoma; Same Origin, Very Distinct Tumor Types. Cancers.

[163] Homsi J., Bedikian A., Papadopoulos N.E., Kim K.B., Hwu W.-J., Mahoney S.L., Hwu P. (2010). Phase 2 open-label study of weekly docosahexaenoic acid–paclitaxel in patients with metastatic uveal melanoma. Melanoma Res..

[164] Kivelä T.T., Suciu S., Hansson J., Kruit W.H.J., Vuoristo M.-S., Kloke O., Gore M., Hahka-Kemppinen M., Parvinen L.-M., Kumpulainen E. (2003). Bleomycin, vincristine, lomustine and dacarbazine (BOLD) in combination with recombinant interferon alpha-2b for metastatic uveal melanoma. Eur. J. Cancer.

[165] Schmittel A., Scheulen M.E., Bechrakis N.E., Strumberg D., Baumgart J., Bornfeld N., Foerster M.H., Thiel E., Keilholz U. (2005). Phase II trial of cisplatin, gemcitabine and treosulfan in patients with metastatic uveal melanoma. Melanoma Res..

[166] Leyvraz S., Piperno-Neumann S. (2014). Hepatic intra-arterial versus intravenous fotemustine in patients with liver metastases from uveal melanoma (EORTC 18021): A multicentric randomized. Ann. Oncol..

[167] Pingpank J.F., Hughes M.S., Alexander H.R., Faries M.B., Zager J.S., Royal R., Whitman E.D., Nutting C.W., Siskin G.P., Agarwala S.S. (2010). A phase III random assignment trial comparing percutaneous hepatic perfusion with melphalan (PHP-mel) to standard of care for patients with hepatic metastases from metastatic ocular or cutaneous melanoma. J. Clin. Oncol..

[168] Gonsalves C.F., Eschelman D.J., Sullivan K.L., Anne P.R., Doyle L., Sato T. (2011). Radioembolization as Salvage Therapy for Hepatic Metastasis of Uveal Melanoma: A Single-Institution Experience. Am. J. Roentgenol..

[169] Klingenstein A., Haug A., Zech C.J., Schaller U.C. (2012). Radioembolization as Locoregional Therapy of Hepatic Metastases in Uveal Melanoma Patients. Cardiovasc. Interv. Radiol..

[170] Bedikian A., Papadopoulos N., Plager C., Eton O., Ring S. (2003). Phase II evaluation of temozolomide in metastatic choroidal melanoma. Melanoma Res..

[171] Spagnolo F., Grosso M., Picasso V., Tornari E., Pesce M., Queirolo P. (2013). Treatment of metastatic uveal melanoma with intravenous fotemustine. Melanoma Res..

[172] O’Neill P., Butt M., Eswar C., Gillis P., Marshall E. (2006). A prospective single arm phase II study of dacarbazine and treosulfan as first-line therapy in metastatic uveal melanoma. Melanoma Res..

[173] Schinzari G., Rossi E., Cassano A., Dadduzio V., Quirino M., Pagliara M., Blasi M.A., Barone C. (2017). Cisplatin, dacarbazine and vinblastine as first line chemotherapy for liver metastatic uveal melanoma in the era of immunotherapy: A single institution phase II study. Melanoma Res..

[174] Khoja L., Atenafu E.G., Suciu S., Leyvraz S., Sato T., Marshall E., Keilholz U., Zimmer L., Patel S., Piperno-Neumann S. (2019). Meta-analysis in metastatic uveal melanoma to determine progression free and overall survival benchmarks: An international rare cancers initiative (IRCI) ocular melanoma study. Ann. Oncol..

[175] Mariani P., Piperno-Neumann S., Servois V., Berry M., Dorval T., Plancher C., Couturier J., Levy-Gabriel C., Rouic L.L.-L., Desjardins L. (2009). Surgical management of liver metastases from uveal melanoma: 16 years’ experience at the Institut Curie. Eur. J. Surg. Oncol. (EJSO).

[176] Frenkel S., Nir I., Hendler K., Lotem M., Eid A., Jurim O., Pe’Er J. (2009). Long-term survival of uveal melanoma patients after surgery for liver metastases. Br. J. Ophthalmol..

[177] Akyuz M., Yazici P., Dural A.C., Yigitbas H., Okoh A., Bucak E., McNamara M., Singh A., Berber E. (2015). Laparoscopic management of liver metastases from uveal melanoma. Surg. Endosc..

[178] Rivoire M., Kodjikian L., Baldo S., Kaemmerlen P., Négrier S., Grange J.-D. (2005). Treatment of Liver Metastases From Uveal Melanoma. Ann. Surg. Oncol..

[179] Augsburger J.J., Correa Z.M., Shaikh A.H. (2009). Effectiveness of Treatments for Metastatic Uveal Melanoma. Am. J. Ophthalmol..

[180] Ripley R.T., Davis J.L., Klapper J.A., Mathur A., Kammula U., Royal R.E., Yang J.C., Sherry R.M., Hughes M.S., Libutti S.K. (2009). Liver Resection for Metastatic Melanoma with Postoperative Tumor-Infiltrating Lymphocyte Therapy. Ann. Surg. Oncol..

[181] Salmon R., Levy C., Plancher C., Dorval T., Desjardins L., Leyvrazi S., Pouillart P., Schlienger P., Servois V., Asselain B. (1998). Treatment of liver metastases from uveal melanoma by combined surgery—chemotherapy. Eur. J. Surg. Oncol. (EJSO).

[182] Aoyama T., Mastrangelo M.J., Berd D., Nathan F.E., Shields C.L., Shields J.A., Rosato E.L., Rosato F.E., Sato T. (2000). Protracted survival after resection of metastatic uveal melanoma. Cancer.

[183] Pawlik T.M., Zorzi D., Abdalla E.K., Clary B. (2006). Hepatic Resection for Metastatic Melanoma: Distinct Patterns of Recurrence and Prognosis for Ocular Versus Cutaneous Disease Endomicroscopy View project geografical disparities in liver transplant View project. Ann. Surg. Oncol..

[184] Adam R., Chiche L., Aloia T., Elias D., Salmon R., Rivoire M., Jaeck D., Saric J., Le Treut Y.P., Belghiti J. (2006). Hepatic resection for noncolorectal nonendocrine liver metastases: Analysis of 1452 patients and development of a prognostic model. Ann. Surg..

[185] de Ridder J.A.M. (2017). Liver Metastases Incidence, Treatment & Prognostic Factors. Ph.D. Thesis.

[186] Groeschl R.T., Nachmany I., Steel J.L., Reddy S.K., Glazer E.S., De Jong M.C., Pawlik T.M., Geller D.A., Tsung A., Marsh J.W. (2012). Hepatectomy for Noncolorectal Non-Neuroendocrine Metastatic Cancer: A Multi-Institutional Analysis. J. Am. Coll. Surg..

[187] Mariani P., Almubarak M.M., Kollen M., Wagner M., Plancher C., Audollent R., Piperno-Neumann S., Cassoux N., Servois V. (2016). Radiofrequency ablation and surgical resection of liver metastases from uveal melanoma. Eur. J. Surg. Oncol. (EJSO).

[188] Derek E., Matsuoka L., Alexopoulos S., Fedenko A.A., Genyk Y., Selby R. (2012). Combined surgical resection and radiofrequency ablation as treatment for metastatic ocular melanoma. Surg. Today.

[189] Servois V., Bouhadiba T., Dureau S., Da Costa D., Almubarak M.M., Foucher R., Savignoni A., Cassoux N., Pierron G., Mariani P. (2019). Iterative treatment with surgery and radiofrequency ablation of uveal melanoma liver metastasis: Retrospective analysis of a series of very long-term survivors. Eur. J. Surg. Oncol..

[190] Olofsson Bagge R., Cahlin C., All-Ericsson C., Hashimi F. (2013). Isolated Hepatic Perfusion for Ocular Melanoma Metastasis: Registry Data Suggests a Survival Benefit Reducing radiation-induced side effects in organs at risk View project PDX v2.0 View project. Artic. Ann. Surg. Oncol..

[191] Search of: NCT01785316—List Results—ClinicalTrials.gov. NCT01785316.

[192] Search of: NCT02678572—List Results—ClinicalTrials.gov. NCT02678572.

[193] Bedikian A.Y., Legha S.S., Mavligit G., Carrasco C.H., Khorana S., Plager C., Papadopoulos N., Benjamin R.S. (1995). Treatment of uveal melanoma metastatic to the liver. A review of the M. D. Anderson cancer center experience and prognostic factors. Cancer.

[194] Feun L.G., Reddy K.R., Yrizarry J.M., Savaraj N., Guerra J.J., Purser R.K., Waldman S., Levi J.U., Moffatt F., Morrell L. (1994). A Phase I Study of Chemoembolization with Cisplatin and Lipiodol for Primary and Metastatic Liver Cancer. Am. J. Clin. Oncol..

[195] Mavligit G., Charnsangavej C., Carrasco C., Jama Y.P., Benjamin R.S., Wallace S. (1988). Regression of ocular melanoma metastatic to the liver after hepatic arterial chemoembolization with cisplatin and polyvinyl sponge. JAMA.

[196] Patel S.P., Kim K.B., Papadopoulos N.E., Hwu W.-J., Hwu P., Prieto V.G., Bar-Eli M., Zigler M., Dobroff A., Bronstein Y. (2011). A phase II study of gefitinib in patients with metastatic melanoma. Melanoma Res..

[197] Gupta S., Bedikian A., Ahrar J., Ensor J., Ahrar K., Madoff D.C., Wallace M.J., Murthy R., Tam A., Hwu P. (2010). Hepatic artery chemoembolization in patients with ocular melanoma metastatic to the liver: Response, survival, and prognostic factors. Am. J. Clin. Oncol..

[198] Vogl T.J., Eichler K., Zangos S., Herzog C., Hammerstingl R., Balzer J., Gholami A. (2006). Preliminary experience with transarterial chemoembolization (TACE) in liver metastases of uveal malignant melanoma: Local tumor control and survival. J. Cancer Res. Clin. Oncol..

[199] Huppert P., Fierlbeck G., Pereira P.L., Schanz S., Duda S., Wietholtz H., Rozeik C., Claussen C.D. (2010). Transarterial chemoembolization of liver metastases in patients with uveal melanoma. Eur. J. Radiol..

[200] Agarwala S., Panikkar R., Kirkwood J.M. (2004). Phase I/II randomized trial of intrahepatic arterial infusion chemotherapy with cisplatin and chemoembolization with cisplatin and polyvinyl sponge in patients with. Melanoma Res..

[201] Gonsalves C.F., Eschelman D.J., Adamo R.D., Anne P.R., Orloff M.M., Terai M., Hage A.N., Yi M., Chervoneva I., Sato T. (2019). A Prospective Phase II Trial of Radioembolization for Treatment of Uveal Melanoma Hepatic Metastasis. Radiology.

[202] Arulananda S., Parakh S., Palmer J., Goodwin M., Andrews M.C., Cebon J. (2019). A pilot study of intrahepatic yttrium-90 microsphere radioembolization in combination with intravenous cisplatin for uveal melanoma liver-only metastases. Cancer Rep..

[203] Search of: NCT01893099—List Results—ClinicalTrials.gov. NCT01893099.

[204] Valsecchi M., Terai M., Eschelman D.J., Gonsalves C.F., Chervoneva I., Shields J.A.P., Shields C.L., Yamamoto A., Sullivan K.L., Laudadio M. (2015). Double-Blinded, Randomized Phase II Study Using Embolization with or without Granulocyte–Macrophage Colony-Stimulating Factor in Uveal Melanoma with. J. Vasc. Interv. Radiol..

[205] Hodi F.S., O’Day S.J., McDermott D.F., Weber R.W., Sosman J.A., Haanen J.B., Gonzalez R., Robert C., Schadendorf D., Hassel J.C. (2010). Improved Survival with Ipilimumab in Patients with Metastatic Melanoma. N. Engl. J. Med..

[206] Garon E.B., Rizvi N.A., Hui R., Leighl N., Balmanoukian A.S., Eder J.P., Patnaik A., Aggarwal C., Gubens M., Horn L. (2015). Pembrolizumab for the Treatment of Non–Small-Cell Lung Cancer. N. Engl. J. Med..

[207] Brahmer J., Reckamp K.L., Baas P., Crino L., Eberhardt W.E., Poddubskaya E., Antonia S., Pluzanski A., Vokes E.E., Holgado E. (2015). Nivolumab versus Docetaxel in Advanced Squamous-Cell Non–Small-Cell Lung Cancer. N. Engl. J. Med..

[208] Zimmer L., Vaubel J., Mohr P., Hauschild A., Utikal J., Šimon J., Garbe C., Herbst R., Enk A., Kämpgen E. (2015). Phase II DeCOG-Study of Ipilimumab in Pretreated and Treatment-Naïve Patients with Metastatic Uveal Melanoma. PLoS ONE.

[209] Rodriguez J.M.P., De Olza M.O., Codes M., Lopez-Martin J.A., Berrocal A., García M., Gurpide A., Homet B., Martin-Algarra S. (2014). Phase II study evaluating ipilimumab as a single agent in the first-line treatment of adult patients (Pts) with metastatic uveal melanoma (MUM): The GEM-1 trial. J. Clin. Oncol..

[210] Joshua A.M., Monzon J.G., Mihalcioiu C., Hogg D., Smylie M., Cheng T. (2015). A phase 2 study of tremelimumab in patients with advanced uveal melanoma. Melanoma Res..

[211] Algazi A.P., Tsai K.K., Shoushtari A.N., Munhoz R.R., Eroglu Z., Piulats J.M., Ott P.A., Johnson D.B., Hwang J., Daud A.I. (2016). Clinical outcomes in metastatic uveal melanoma treated with PD-1 and PD-L1 antibodies. Cancer.

[212] Mignard C., Huvier A.D., Gillibert A., Modeste A.B.D., Dutriaux C., Khammari A., Avril M.-F., Kramkimel N., Mortier L., Marcant P. (2018). Efficacy of Immunotherapy in Patients with Metastatic Mucosal or Uveal Melanoma. J. Oncol..

[213] Johnson D.B., Bao R., Ancell K.K., Daniels A.B., Wallace D., Sosman J.A., Luke J.J. (2019). Response to Anti–PD-1 in Uveal Melanoma Without High-Volume Liver Metastasis. J. Natl. Compr. Cancer Netw..

[214] Rodrigues M., Mobuchon L., Houy A., Fiévet A., Gardrat S., Barnhill R.L., Popova T., Servois V., Rampanou A., Mouton A. (2018). Outlier response to anti-PD1 in uveal melanoma reveals germline MBD4 mutations in hypermutated tumors. Nat. Commun..

[215] Search of: NCT02626962—List Results—ClinicalTrials.gov. NCT02626962.

[216] Search of: NCT01585194—List Results—ClinicalTrials.gov. NCT01585194.

[217] Pelster M., Gruschkus S.K., Bassett R., Gombos D.S., Shephard M., Posada L., Glover M., Diab A., Hwu P., Patel S.P. (2019). Phase II study of ipilimumab and nivolumab (ipi/nivo) in metastatic uveal melanoma (UM). J. Clin. Oncol..

[218] Search of: NCT02697630—List Results—ClinicalTrials.gov. NCT02697630.

[219] Jespersen H., Bagge R.O., Ullenhag G., Carneiro A., Helgadottir H., Ljuslinder I., Levin M., All-Eriksson C., Andersson B., Stierner U. (2019). Concomitant use of pembrolizumab and entinostat in adult patients with metastatic uveal melanoma (PEMDAC study): Protocol for a multicenter phase II open label study. BMC Cancer.

[220] Rozeman E.A., Prevoo W., Meier M.A., Sikorska K., Van T.M., Van De Wiel B.A., Van Der Wal J.E., Mallo H.A., Grijpink-Ongering L.G., Broeks A. (2020). Phase Ib/II trial testing combined radiofrequency ablation and ipilimumab in uveal melanoma (SECIRA-UM). Melanoma Res..

[221] Search of: NCT04283890—List Results—ClinicalTrials.gov. NCT04283890.

[222] Search of: NCT03472586—List Results—ClinicalTrials.gov. NCT03472586.

[223] Search of: NCT02913417—List Results—ClinicalTrials.gov. NCT02913417.

[224] Chandran S., Somerville R., Yang J., Sherry R.M., Klebanoff C.A., Goff S.L., Wunderlich J.R., Danforth D.N., Zlott D., Paria B.C. (2017). Treatment of metastatic uveal melanoma with adoptive transfer of tumour-infiltrating lymphocytes: A single-centre, two-stage, single-arm, phase 2 study. Lancet Oncol..

[225] van Loenen M.M., de Boer R., Hagedoorn R.S., Jankipersadsing V., Amir A.L., Falkenburg J.H.F., Heemskerk M.H.M. (2013). Multi-cistronic vector encoding optimized safety switch for adoptive therapy with T-cell receptor-modified T cells. Gene Ther..

[226] Forsberg E.M.V., Lindberg M.F., Jespersen H., Alsén S., Bagge R.O., Donia M., Svane I.M., Nilsson O., Ny L., Nilsson L.M. (2019). HER2 CAR-T Cells Eradicate Uveal Melanoma and T-cell Therapy–Resistant Human Melanoma in IL2 Transgenic NOD/SCID IL2 Receptor Knockout Mice. Cancer Res..

[227] Tavera R.J., Forget M.A., Kim Y.U., Sakellariou-Thompson D., Creasy C.A., Bhatta A., Fulbright O.J., Ramachandran R., Thorsen S.T., Flores E. (2018). Utilizing T-cell Activation Signals 1, 2, and 3 for Tumor-infiltrating Lymphocytes (TIL) Expansion: The Advantage over the Sole Use of Interleukin-2 in Cutaneous and Uveal Melanoma. J. Immunother..

[228] Search of: NCT03467516—List Results—ClinicalTrials.gov. NCT03467516.

[229] Search of: NCT03068624—List Results—ClinicalTrials.gov. NCT03068624.

[230] Damato B., Dukes J., Goodall H., Carvajal R.D. (2019). Tebentafusp: T Cell Redirection for the Treatment of Metastatic Uveal Melanoma. Cancers.

[231] Middleton M., Steven N.M., Evans T.J., Infante J.R., Sznol M., Mulatero C., Hamid O., Shoushtari A.N., Shingler W., Johnson A. (2016). Safety, pharmacokinetics and efficacy of IMCgp100, a first-in-class soluble TCR-antiCD3 bispecific t cell redirector with solid tumour activity: Results from the FIH study in melanoma. J. Clin. Oncol..

[232] Search of: NCT02570308—List Results—ClinicalTrials.gov. NCT02570308.

[233] Search of: NCT03070392—List Results—ClinicalTrials.gov. NCT03070392.

[234] Search of: NCT03635632—List Results—ClinicalTrials.gov. NCT03635632.

[235] Bol K.F., Mensink H.W., Aarntzen E.H., Schreibelt G., Keunen J.E., Coulie P.G., De Klein A., Punt C.J., Paridaens D., Figdor C.G. (2014). Long Overall Survival After Dendritic Cell Vaccination in Metastatic Uveal Melanoma Patients. Am. J. Ophthalmol..

[236] Sarnaik A.A., Yu B., Yu D., Morelli D., Hall M., Bogle D., Yan L., Targan S., Solomon J., Nichol G. (2010). Extended Dose Ipilimumab with a Peptide Vaccine: Immune Correlates Associated with Clinical Benefit in Patients with Resected High-Risk Stage IIIc/IV Melanoma. Clin. Cancer Res..

[237] Lesterhuis W.J., Schreibelt G., Scharenborg N.M., Brouwer H.M.H., Gerritsen M.P., Croockewit S., Coulie P.G., Torensma R., Adema G.J., Figdor C.G. (2011). Wild-type and modified gp100 peptide-pulsed dendritic cell vaccination of advanced melanoma patients can lead to long-term clinical responses independent of the peptide used. Cancer Immunol. Immunother..

[238] Bosch J.J., Iheagwara U.K., Reid S., Srivastava M.K., Wolf J., Lotem M., Ksander B.R., Ostrand-Rosenberg S. (2009). Uveal melanoma cell-based vaccines express MHC II molecules that traffic via the endocytic and secretory pathways and activate CD8+ cytotoxic, tumor-specific T cells. Cancer Immunol. Immunother..

[239] Thompson J.F., Agarwala S.S., Smithers B., Ross M.I., Scoggins C.R., Coventry B.J., Neuhaus S.J., Minor D.R., Singer J.M., Wachter E.A. (2014). Phase 2 Study of Intralesional PV-10 in Refractory Metastatic Melanoma. Ann. Surg. Oncol..

[240] Search of: NCT00219843—List Results—ClinicalTrials.gov. NCT00219843.

[241] Van Raamsdonk C.D., Bezrookove V., Green G., Bauer J., Gaugler L., O’Brien J.M., Simpson E.M., Barsh G.S., Bastian B.C. (2008). Frequent somatic mutations of GNAQ in uveal melanoma and blue naevi. Nature.

[242] Van Raamsdonk C.D., Griewank K., Crosby M.B., Garrido M.C., Vemula S., Wiesner T., Obenauf A.C., Wackernagel W., Green G., Bouvier N. (2010). Mutations inGNA11in Uveal Melanoma. N. Engl. J. Med..

[243] Robert C., Grob J.J., Stroyakovskiy D., Karaszewska B., Hauschild A., Levchenko E., Chiarion-Sileni V., Schachter J., Garbe C., Bondarenko I. (2019). Five-Year Outcomes with Dabrafenib plus Trametinib in Metastatic Melanoma. N. Engl. J. Med..

[244] Ascierto P.A., Dummer R., Gogas H.J., Flaherty K.T., Arance A., Mandala M., Liszkay G., Garbe C., Schadendorf D., Krajsova I. (2020). Update on tolerability and overall survival in COLUMBUS: Landmark analysis of a randomised phase 3 trial of encorafenib plus binimetinib vs vemurafenib or encorafenib in patients with BRAF V600-mutant melanoma. Eur. J. Cancer.

[245] Onken M., Makepeace C. (2018). Targeting nucleotide exchange to inhibit constitutively active G protein α subunits in cancer cells. Sci. Signal..

[246] Lapadula D., Farias E., Randolph C.E., Purwin T.J., McGrath D., Charpentier T.H., Zhang L., Wu S., Terai M., Sato T. (2018). Effects of Oncogenic Gαq and Gα11 Inhibition by FR900359 in Uveal Melanoma. Mol. Cancer Res..

[247] Xiong X.-F., Zhang H., Boesgaard M.W., Underwood C.R., Bräuner-Osborne H., Strømgaard K. (2019). Structure–Activity Relationship Studies of the Natural Product G q/11 Protein Inhibitor YM-254890. ChemMedChem.

[248] Li Y., He J., Qiu C., Shang Q., Qian G., Fan X., Ge S., Jia R. (2018). The oncolytic virus H101 combined with GNAQ siRNA-mediated knockdown reduces uveal melanoma cell viability. J. Cell. Biochem..

[249] Posch C., Latorre A., Crosby M.B., Celli A., Latorre A., Vujic I., Sanlorenzo M., Green G.A., Weier J., Zekhtser M. (2015). Detection of GNAQ mutations and reduction of cell viability in uveal melanoma cells with functionalized gold nanoparticles. Biomed. Microdevices.

[250] Carvajal R.D., Piperno-Neumann S., Kapiteijn E., Chapman P.B., Frank S., Joshua A.M., Piulats J.M., Wolter P., Cocquyt V., Chmielowski B. (2018). Selumetinib in combination with dacarbazine in patients with metastatic uveal melanoma: A phase III, multicentre, randomised trial (SUMIT). J. Clin. Oncol..

[251] Ambrosini G., Musi E., Ho A.L., De Stanchina E., Schwartz G.K. (2013). Inhibition of Mutant GNAQ Signaling in Uveal Melanoma Induces AMPK-Dependent Autophagic Cell Death. Mol. Cancer Ther..

[252] Search of: NCT01979523—List Results—ClinicalTrials.gov. NCT01979523.

[253] Bhatia S., Moon J., Margolin K.A., Weber J.S., Lao C.D., Othus M., Aparicio A.M., Ribas A., Sondak V.K. (2012). Phase II Trial of Sorafenib in Combination with Carboplatin and Paclitaxel in Patients with Metastatic Uveal Melanoma: SWOG S0512. PLoS ONE.

[254] Search of: NCT02601378—List Results—ClinicalTrials.gov. NCT02601378.

[255] Zeldis J., Heller C., Seidel G., Yuldasheva N., Stirling D., ShutackS Y., Libutti K. (2009). A randomized phase II trial comparing two doses of lenalidomide for the treatment of stage IV ocular melanoma. J. Clin. Oncol..

[256] Luke J.J., Olson D.J., Allred J.B., Strand C.A., Bao R., Zha Y., Carll T., Labadie B.W., Bastos B.R., Butler M.O. (2019). Randomized Phase II Trial and Tumor Mutational Spectrum Analysis from Cabozantinib versus Chemotherapy in Metastatic Uveal Melanoma (Alliance A091201). Clin. Cancer Res..

[257] Search of: NCT01413191—List Results—ClinicalTrials.gov. NCT01413191.

[258] Search of: NCT01252251—List Results—ClinicalTrials.gov. NCT01252251.

[259] Shoushtari A.N., Ong L.T., Schoder H., Singh-Kandah S., Abbate K.T., Postow M.A., Callahan M.K., Wolchok J., Chapman P.B., Panageas K.S. (2016). A phase 2 trial of everolimus and pasireotide long-acting release in patients with metastatic uveal melanoma. Melanoma Res..

[260] Daud A.I., Kluger H.M., Kurzrock R., Schimmoller F., Weitzman A.L., Samuel T.A., Moussa A.H., Gordon M.S., Shapiro G. (2017). I Phase II randomised discontinuation trial of the MET/VEGF receptor inhibitor cabozantinib in metastatic melanoma. Br. J. Cancer.

[261] Shah S., Luke J.J., Jacene H.A., Chen T., Giobbie-Hurder A., Ibrahim N., Buchbinder E.I., McDermott D.F., Flaherty K.T., Sullivan R.J. (2018). Results from phase II trial of HSP90 inhibitor, STA-9090 (ganetespib), in metastatic uveal melanoma. Melanoma Res..

[262] Guenterberg K., Grignol V., ROlenckielekar K.V., Varker K.A., Chen H.X., Kendra K.L., Olencki T.L., Carson W.E. (2011). A pilot study of bevacizumab and interferon-α2b in ocular melanoma. Am. J. Clin. Oncol..

[263] Piperno-Neumann S., Servois V., Bidard F.-C., Mariani P., Plancher C., Diallo A., Vago-Ady N., Desjardins L. (2013). BEVATEM: Phase II study of bevacizumab (B) in combination with temozolomide (T) in patients (pts) with first-line metastatic uveal melanoma (MUM): Final results. J. Clin. Oncol..

[264] Spitler L.E., Boasberg P., Day S.O., Hamid O., Cruickshank S., Mesko S., Weber R.W. (2015). Phase II Study of Nab-Paclitaxel and Bevacizumab as First-line Therapy for Patients with Unresectable Stage III and IV Melanoma. Am. J. Clin. Oncol..

[265] Tarhini A.A., Frankel P., Margolin K.A., Christensen S., Ruel C., Shipe-Spotloe J., Gandara D.R., Chen A., Kirkwood J.M. (2011). Aflibercept (VEGF Trap) in Inoperable Stage III or Stage IV Melanoma of Cutaneous or Uveal Origin. Clin. Cancer Res..

[266] Hofmann U.B., Kauczok-Vetter C.S., Houben R., Becker J.C. (2009). Overexpression of the KIT/SCF in Uveal Melanoma Does Not Translate into Clinical Efficacy of Imatinib Mesylate. Clin. Cancer Res..

[267] Penel N., Delcambre C., Durando X., Clisant S., Hebbar M., Négrier S., Fournier C., Isambert N., Mascarelli F., Mouriaux F. (2008). O-Mel-Inib: A Cancéro-pôle Nord-Ouest multicenter phase II trial of high-dose Imatinib mesylate in metastatic uveal melanoma. Investig. New Drugs.

[268] Mahipal A., Tijani L., Chan K., Laudadio M., Mastrangelo M.J., Sato T. (2012). A pilot study of sunitinib malate in patients with metastatic uveal melanoma. Melanoma Res..

[269] Haas N.B., Quirt I., Hotte S., McWhirter E., Polintan R., Litwin S., Adams P.D., McBryan T., Wang L., Martin L.P. (2014). Phase II trial of vorinostat in advanced melanoma. Investig. New Drugs.

[270] Search of: NCT03417739—List Results—ClinicalTrials.gov. NCT03417739.

[271] Search of: NCT02743611—List Results—ClinicalTrials.gov. NCT02743611.

[272] Search of: NCT01835145—List Results—ClinicalTrials.gov. NCT01835145.

